# Evaluation of Frequency of CMV Replication and Disease Complications Reveals New Cellular Defects and a Time Dependent Pattern in CVID Patients

**DOI:** 10.1007/s10875-024-01744-3

**Published:** 2024-06-07

**Authors:** Luca Marri, Paola Contini, Federico Ivaldi, Chiara Schiavi, Ottavia Magnani, Chiara Vassallo, Andrea Guastalla, Noemi Traversone, Claudia Angelini, Genny Del Zotto, Andrea De Maria, Raffaele De Palma

**Affiliations:** 1 Department of Internal Medicine (DIMI), University of Genoa, Genoa, Italy; 2 Unit of Clinical Immunology and Translational Medicine, IRCCS Ospedale Policlinico San Martino, Genoa, Italy; 3 Institute for Applied Mathematics “Mauro Picone”, National Research Council, Naples, Italy; 4 Integrated Department of Services and Laboratories, IRCCS Istituto Giannina Gaslini, Genoa, Italy; 5 Department of Health Sciences (DISSAL), University of Genoa, Genoa, Italy; 6 Infections in Immunocompromised Host Unit, IRCCS Ospedale Policlinico San Martino, Genoa, Italy; 7 Department of Experimental Medicine (DIMES), University of Genoa, Genoa, Italy

**Keywords:** CVID, Humoral immunodeficiencies, CMV, EBV, Inflammatory lymphoid precursors, Clinical phenotypes

## Abstract

**Purpose:**

Common Variable Immunodeficiency (CVID) is characterized by hypogammaglobulinemia and failure of specific antibody production due to B-cell defects. However, studies have documented various T-cell abnormalities, potentially linked to viral complications. The frequency of Cytomegalovirus (CMV) replication in CVID cohorts is poorly studied. To address this gap in knowledge, we set up an observational study with the objectives of identifying CVID patients with active viraemia (CMV, Epstein-Barr virus (EBV)), evaluating potential correlations with immunophenotypic characteristics, clinical outcome, and the dynamic progression of clinical phenotypes over time.

**Methods:**

31 CVID patients were retrospectively analysed according to viraemia, clinical and immunologic characteristics. 21 patients with non CVID humoral immunodeficiency were also evaluated as control.

**Results:**

Active viral replication of CMV and/or EBV was observed in 25% of all patients. CMV replication was detected only in CVID patients (16%). CVID patients with active viral replication showed reduced HLA-DR^+^ NK counts when compared with CMV-DNA negative CVID patients. Viraemic patients had lower counts of LIN^−^DNAM^bright^ and LIN^−^CD16^+^ inflammatory lymphoid precursors which correlated with NK-cell subsets. Analysis of the dynamic progression of CVID clinical phenotypes over time, showed that the initial infectious phenotype progressed to complicated phenotypes with time. All CMV viraemic patients had complicated disease.

**Conclusion:**

Taken together, an impaired production of inflammatory precursors and NK activation is present in CVID patients with active viraemia. Since “Complicated” CVID occurs as a function of disease duration, there is need for an accurate evaluation of this aspect to improve classification and clinical management of CVID patients.

**Supplementary Information:**

The online version contains supplementary material available at 10.1007/s10875-024-01744-3.

## Introduction

Common Variable Immunodeficiency Disorders (CVID) represent the most prevalent category of clinically significant primary immunodeficiency disorders, estimated to affect up to 1 in 25,000 individuals [1, 2]. CVID includes a heterogeneous spectrum of disorders characterized by the disruption of B cell differentiation, resulting in impaired immunoglobulin production with heterogeneous immunophenotype. This variability in CVID is characterized by an extensive array of clinical manifestations, including recurrent infections sustained by extracellular pathogens, chronic respiratory diseases, autoimmune disorders, gastrointestinal involvement, and an increased susceptibility to developing lymphomas and solid tumors [1–7]. Over the last decade, a substantial number of patients have been shown to have dysregulated conventional and regulatory T-cells in addition to B cell deficiencies [5, 8–16]. Accordingly, the most commonly used prognostic classifications [17–19], based on the B cell immunophenotype determination are insufficient for patient classification/prognosis.

In addition, an increased susceptibility to Cytomegalovirus (CMV) reactivation and disease has been surprisingly documented in some CVID patients. Severe CMV infection, albeit rare, has a significant impact on patient prognosis and mortality, as reflected by several case reports on a total of 40 patients [20, 21]. Furthermore, CVID patients with latent chronic CMV infection have been proposed to present an increased risk of developing autoimmune or inflammatory manifestations, and that this may be hypothetically linked to the presence of a dysregulation of cellular responses and CMV-specific T-cells [22–24].

In the context of CMV infection, in addition to CMV-specific T-cells, control of CMV has been also associated with NK-cells [25–28] and with two distinct peripheral blood precursor populations (CD34^+^DNAM-1^bright^CXCR4^+^ and Lin^−^CD56^−^CD16^+^Perf^−^CD94^−^CXCR4^+^ precursors) that are released from the bone marrow and generate in vitro high functional NK-cells [29, 30]. So far there is no information on these cells in CVID patients.

Moreover, an additional area of uncertainty still pertains the relationship between complicated disease and dynamic progression of CVID clinical phenotypes over time, from onset to last clinical evaluation [3, 6, 31–34].

In view of the still open questions whether clinical severity may reflect disease duration and may be linked to frequency of CMV replication and whether there may be an involvement of T-cells, NK-cells or inflammatory precursors, we performed an observational study in a cohort of CVID patients with over 13 years follow-up.

Here, we show that a proportion of CVID patients in the cohort fails to control CMV replication, showing less inflammatory precursors and activated NK-cells. In addition, we provide evidence that complicated disease occurs as a function of time on follow-up with progressive accrual of symptoms in patients over time. These results may contribute to improve the classification and follow-up of CVID patients.

## Materials and Methods

### Study Design

This retrospective/cross-sectional, monocentric, no profit study was conducted in the Internal Medicine Clinical Immunology and Translational Medicine Department of San Martino Hospital, in Genoa. A primary objective of this study was to analyse the prevalence of CMV and Epstein-Barr virus (EBV) blood replication in a cohort of CVID patients and its impact on clinical and immunophenotypic parameters including the evaluation of circulating inflammatory lymphoid precursors. A secondary objective was to analyse the dynamic progression of CVID clinical phenotypes over time in order to better understand the relationship between disease duration, “complicated phenotypes” and disease severity.

### Patients and Data Collection

CVID affected patients (#31) were enrolled after providing informed consent. CVID diagnosis was made according to either European Society for Immunodeficiencies (ESID)/ Pan-American Group for Immunodeficiency (PAGID) or International Consensus Document (ICON) diagnostic criteria [35, 36]. Age of onset and disease duration were determined based on the first documented occurrence of hypogammaglobulinemia. In the context of routine investigations, a peripheral blood sample was collected from each patient for the cytometric assays outlined in the study. CMV and EBV DNA was assayed in all patients at the time of flow-cytometric analysis, in order to identify those at risk of progressive disease and not according to clinical and diagnostic needs. We further specify that there was no active screening protocol for detecting CMV and EBV viraemia in CVID patients, making it impossible to ascertain the prior viral status of the patients.

The remaining data were derived from medical records and clinical interviews and subsequently transferred to a fully de-identified database for statistical analysis. The collected variables encompassed sociodemographic and clinical characteristics, as well as laboratory parameters, all of which are comprehensively detailed in Table 1. In our study, patients presenting with any non-infectious manifestations including autoimmunity, granulomatous disease, or malignancy are categorized as “complicated”.

**Table 1:** Clinical and laboratory variables

CLINICAL OUTCOME VARIABLE	
Infections	Presence of at least one of these: - Recurrent sinusitis, otitis, or high respiratory tract infections - Pneumonia - Recurrent non-life-threatening infections (e.g. Erysipelas, cholangitis) - Gastro-intestinal infection caused by CVID-characteristic pathogen (e.g. Campylobacter, Giardia, Norovirus, etc.) - Severe infection (sepsis, meningoencephalitis, endocarditis etc.) - Opportunistic infection
Autoimmunity	Presence of at least one autoimmune manifestation, including both systemic and organ-specific autoimmune diseases. Dysregulated or inflammatory manifestations such as enteropathies and non-infectious erythema nodosum were also included in this group
Granulomatous disease	Histological evidence, with or without radiological confirmation, of granulomatous lesions with no discernible infectious cause
Lung involvement	Presence of at least one of the following: high-resolution tomography alterations, such as bronchiectasis or interstitial abnormalities, documented reduction in DLCO, or a restrictive pattern observed in pulmonary function testing
Malignancies	The diagnosis should be established within 6 months of the CVID diagnosis. Benign solid tumors are not considered
Atopy	At least one allergic manifestation, encompassing a history of anaphylaxis, allergic rhino-conjunctivitis, asthma, and atopic dermatitis
Splenomegaly	Spleen diameter > 12 cm, evaluated by ultrasound or CT scan
Lymphadenopathy (LA)	Chronic enlargement of at least two different lymphnode anatomical sites demonstrated through ultrasound or CT scan
LABORATORY VARIABLES	
Immunoglobulin levels	Total IgG levels before initiation of Ig replacement therapy, as well as total IgA, IgM, and IgE levels at the most recent evaluation, were determined
CMV and EBV blood viral load	CMV viral load was determined by RT-PCR with CMV ELITe MGB Kit EBV viral load was determined by RT-PCR with EBV ELITe MGB Kit
Other infective agents	Each patient underwent screening for HCV, HIV, HBV, HHV6 using RT-PCR tests (in addition, for HIV, a fourth-generation test was also conducted), and anamnestic immune response to MTB

*CMV* Cytomegalovirus; *DLCO* Diffusing Capacity of the Lungs for Carbon Monoxide; *EBV* Epstein-Barr virus; *HBV* Hepatitis B virus; *HCV* hepatitis C virus; *HHV6* Human herpes virus 6; *HIV* human immunodeficiency virus; *MTB* Mycobacterium tuberculosis; *RT-PCR* Real time polymerase chain reaction

**Fig. 1:**
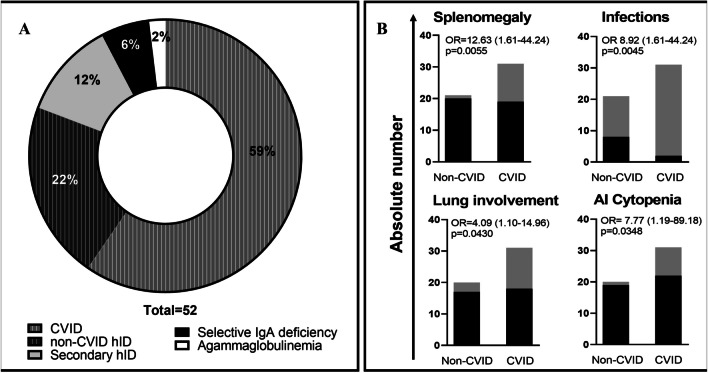
**A**. Humoral immunodeficiency cohort. **B** Clinical differences between CVID and Non-CVID patients. Black bars and gray bars indicate respectively the absence and the presence of the specific clinical manifestation in non-CVID and CVID groups (P values were based on two-sided Fisher’s exact test; odds ratios were reported with 95% CI in parenthesis). Abbreviations: AI: autoimmune; CVID: Common Variable Immunodeficiency Disorders; hID: humoral Immunodeficiency; OR: odds ratio

A control group of 21 patients with non-CVID hID was included and subjected to identical analyses. This group includes patients with selective IgA deficiency, secondary hID, agammaglobulinemia, and patients with primary hypogammaglobulinemia who did not meet the criteria for CVID (Fig. 1A).

For CVID patients, symptomatic disease duration was defined as the time from the onset of symptoms to the time of flow cytometric evaluation. We defined the onset manifestation as the predominant clinical phenotype (e.g., infectious, autoimmune, granulomatous) based on the onset clinical symptoms.

### Flow Cytometric Analyses

Flow cytometric analyses were performed using the Cytoflex LX instrument (Beckman Coulter) with heparinized whole blood, and data were analysed using Kaluza Analysis 2.1 software (antibody panel composition is provided in supplementary materials). There is a singular exception within this study, specifically concerning the B-cell sub-populations. These sub-populations were collected from medical records, having been conducted as part of routine laboratory diagnostic tests for patients with CVID (diagnostic Laboratory utilizing the DxFLEX flow cytometer (Beckman Coulter)).

Leukocyte and conventional lymphocyte populations were identified using a CD45/SSC-gating strategy (supplementary Fig. S1A). The expression of CD69, HLA-DR, and CD38 was assessed in NK-cells and T-cell sub-populations. To evaluate potential lymphoid precursors, a Side Scatter (SSC)/Forward Scatter (FSC) gating strategy was employed to detect lymphocytes, thus including CD45 negative cells (supplementary Fig. S1B). Subsequently, an estimation of LIN^−^DNAM^bright^ precursors was performed since these cells have been shown to be 100% CD34^+^CXCR4^+^ inflammatory precursors [29, 30]. In addition, CD34^−^LIN^−^CD16^+^CD7^−^ precursors, identified here as LIN^−^CD16^+^ with no co-expression of CD45 and CD38, were also estimated. A volumetric beadless absolute count was performed for each cell population.

### Statistical Analysis

Comparisons between the clinical and immunologic categorical variables were conducted through cross tabulations, with statistical significance assessed through two-sided Fisher’s exact test. As for continuous variables, comparisons were conducted employing either parametric (Student's t-test) or non-parametric (Mann–Whitney U test) tests depending on the distribution of the variables and sample size. Correlations were assessed using Spearman's rho coefficient and p values were adjusted according to Benjamini–Hochberg correction. All statistical analyses were conducted using Graphpad Prism version 9.1.0 for Windows (GraphPad Software, San Diego, California USA, www.graphpad.com) and Jamovi version 2.3.28 software [37].

## Results

### Clinical and Immunophenotypic Characterization of CVID Patients Compared to a Non-CVID hID Cohort

In this study, a cohort of 52 hID patients was examined. 31 patients fulfilled the diagnostic criteria for CVID, whereas the remaining 21 participants constituted the non-CVID hID control group (Fig. 1A).

All cases of secondary hID were attributed to the iatrogenic effects of immunosuppressive drugs (rituximab, corticosteroids, cyclophosphamide, methotrexate, and leflunomide) administered for the treatment of either autoimmune conditions (spondylarthritis, rheumatoid arthritis, vasculitis) or hematologic disorders (chronic lymphatic leukaemia).

Clinical and demographic characteristics of CVID and non-CVID hID patients, are reported and compared in Table 2 The two populations were similar in terms of sex distribution, age, disease onset, and duration. Immunoglobulin replacement therapy was significantly more frequent among CVID patients. With respect to clinical presentation phenotypes, the CVID cohort demonstrated a significantly increased prevalence of infections, splenomegaly, pulmonary disorders, and autoimmune cytopenia (Fig. 1B).

**Table 2:** Sample demographics and clinical characteristics

	Total (52)	CVID (31)	non-CVID (21)	OR/ MD	*P* value (univariate)
SAMPLE DEMOGRAPHICS					
Male	21 (40%)	14 (45%)	7 (33%)	1.647	0.5653
Female	31 (60%)	17 (55%)	14 (67%)	0.6071	0.5653
Age	52.4 (48.0–56.8)	50.4 (44.1–56.7)	55.1 (48.9–61.4)	-4.7	0.3110
CLINICAL VARIABLES					
Age at diagnosis	42.25 (36.9–47.6)	39.0 (32.3–45.8)	47.3 (38.4–56.1)	-8.2	0.1308
Disease duration	11.6 (8.0–15.2)	13.7 (8.8–18.5)	8.5 (2.8–14.1)	5.2	0.1481
Ig replacement therapy (IGRT)	32 (62%)	26 (84%)	6 (28%)	13.00	** < 0.0001**
IGRT duration	9.3 (5.5–13.1)	8.1 (4.8–11.4)	14.3 (-4.6–33.2)	-6.2	0.3474
Infections	42 (81%)	29 (94%)	13 (62%)	8.923	**0.0045**
Autoimmunity (AI)	34 (65%)	19 (61%)	15 (71%)	0.6333	0.4508
AI Cytopenia	10 (19%)	9 (29%)	1 (5%)	7.773	**0.0348**
Splenomegaly	13 (25%)	12 (39%)	1 (5%)	12.63	**0.0055**
Granulomatosis	9 (17%)	6 (19%)	3 (14%)	1.440	0.6354
Malignancies	11 (21%)	6 (19%)	5 (24%)	0.7680	0.6995
Lymphadenopathy	16 (31%)	12 (39%)	4 (19%)	2,684	0.1317
Lung involvement	16 (31%)	13 (42%)	3 (15%)	4.093	**0.0430**
Atopy	18 (35%)	10 (32%)	8 (38%)	0.7738	0.6642
Blood CMV replication	5 (10%)	5 (16%)	0 (0%)	Infinity	0.0529
Blood EBV replication	11 (21%)	6 (19%)	5 (24%)	0.7680	0.6995
Blood EBV/CMV replication	13 (25%)	8 (26%)	5 (24%)	1.113	0.8704

Quantitative variables are expressed as means with a 95% confidence interval, while qualitative variables are represented as absolute frequencies with percentages relative to the total of the respective column. P values were calculated by two-sided Fisher test for qualitative variables and by either unpaired two-tailed Student's t-test or Mann–Whitney u test for quantitative variables according to variable distribution and sample size. OR: odds ratio; MD: mean difference

**Fig. 2:**
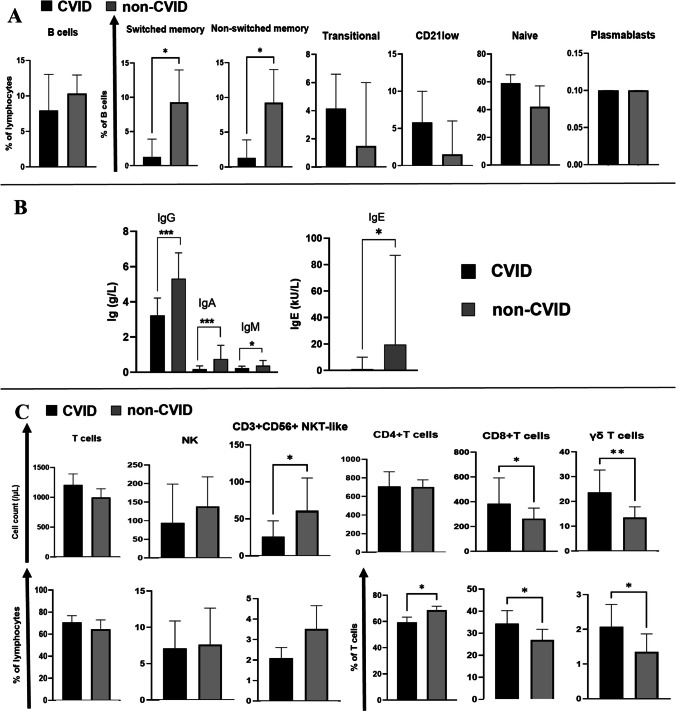
**A** Comparison of peripheral B-cell subset phenotypes between CVID and non-CVID humoral immunodeficiency (hID) patients. Black bars and gray bars indicate respectively CVID and non-CVID hID populations. B cell (CD19^+^ lymphocytes) subests are defined as follow: switched memory B cells = CD27 + CD21 + CD38 + IgM-IgD-; non-switched memory B cells = CD27^+^CD21^+^CD38^+^IgM^++^IgD^+^; CD21^low^ B cells = CD27^−^CD21^low^CD38^low^IgM^+^IgD^+^; Naïve B cells: CD27^−^CD21^+^CD38^+^IgM^+^IgD^++^; Transitional B cells: CD27^−^CD21^+^CD38^++^IgM^++^IgD^++^; Plasmablasts: CD27^++^CD21^+^CD38^+++^IgD^−^ **B** IgG values at onset and IgA, IgM and IgE values at last evaluation are compared between CVID (black bars) and non-CVID hID (gray bars) patients. **C**. Comparison of lymphoid cell populations (T-cells: total, CD4 + , CD8 + , g/d; NK-cells; NKT-like cells) between CVID and non-CVID hID patients. Black bars and gray bars indicate respectively CVID and non-CVID hID populations. The respective lymphoid population are indicated upon each column. Absolute and percentage count are represented respectively in the first and second raw. The histograms show the medians with 95% CI. P values are based on Mann–Whitney U test (*p* < 0,05:*; *p* < 0,01:**; *p* < 0,001:***). Abbreviations: Lymph.: lymphocytes

We next assessed immunological parameters by flow cytometry comparing CVID vs. non-CVID hID patients in order to verify the consistency of diagnosis (Table S1 and Fig. 2).

Accordingly, CVID patients displayed reduced levels of all immunoglobulin isotypes, in addition to a reduction in class-switched memory B cells (CD19^+^IgM^−^IgD^−^ CD27^+^CD21^+^CD38^+^), which is considered an immunophenotypic hallmark of the disease. Notably, in CVID patients, there was a discernible decrease not only in switched memory (sm)-B cells but also in non-sm B cells (CD19^+^IgM^++^IgD^+^CD38^+^CD27^+^CD21^+^), thereby underscoring an impaired B cell activation. In addition, concerning T-cell phenotypes, CVID patients exhibited higher relative and absolute αβ CD8^+^ T-cell counts and a decreased frequency of αβ CD4^+^ T-cells (Fig. 2C), resulting in a reduced CD4/CD8 ratio in CVID vs. non-CVID hID patients (respectively: 1.91 [CI95% 1.56–2.26]; 2.51 [CI95% 2.11–2.91]; p = 0.0181, two-tailed Mann Whitney u test).

Taken together, these data confirmed that CVID patients have a distinct clinical immunophenotypic signature with altered T and NK cell frequencies along with B cell defect, while this is not the case in other ID presenting with hypogammaglobulinemia.

### “Complicated” Clinical Phenotype in CVID Patients is a Function of Disease Duration

**Fig. 3:**
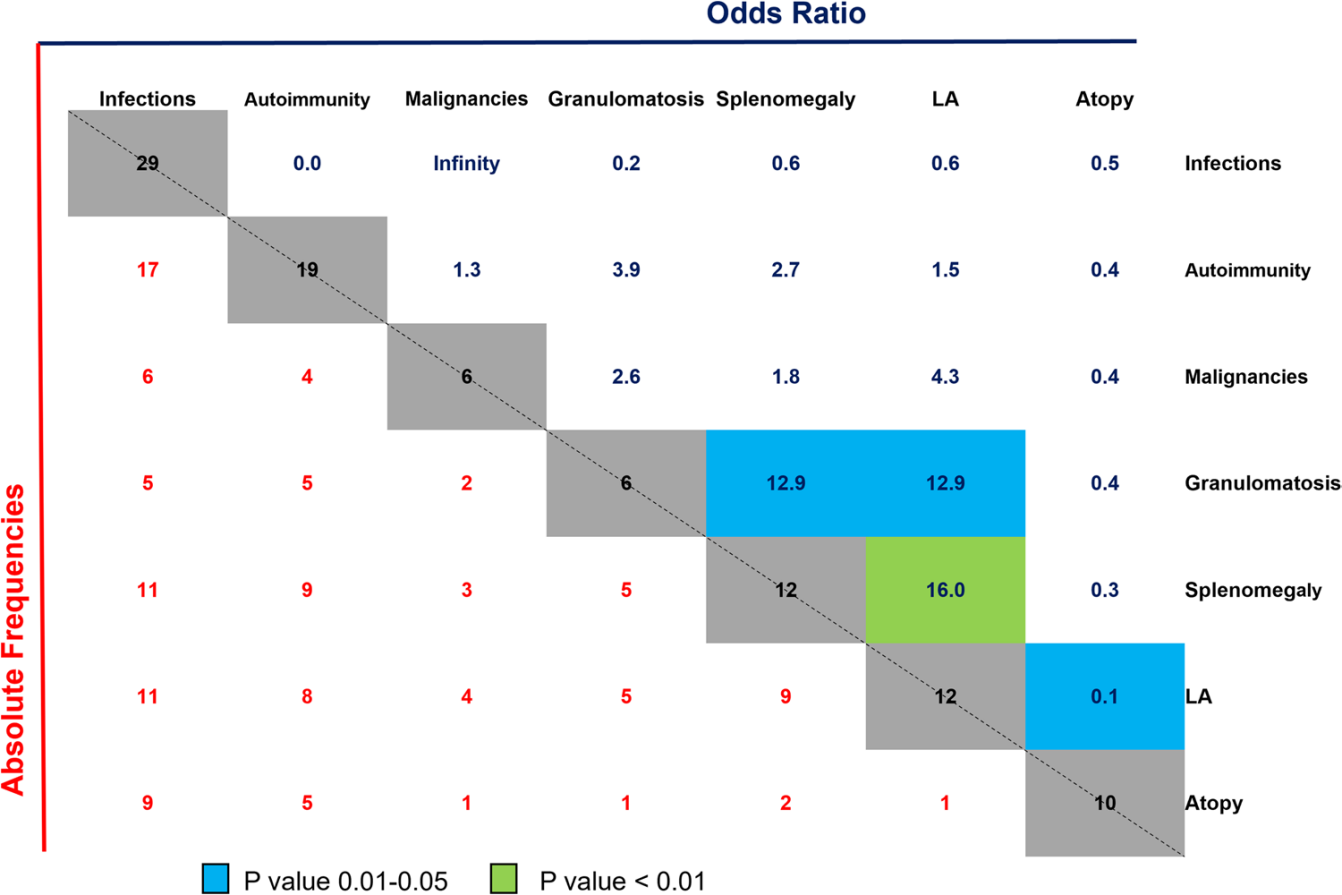
Features of clinical presentation of CVID patients with absolute frequencies and the relative odds ratio of the probability of their occurrences. Numbers in the lower left triangle (red text) indicate the absolute frequencies of occurrence/co-occurrence of symptoms among all CVID patients. Numbers in the upper right triangle (blue text) show the odds ratio for the probability of occurrence of the combined phenotypes. Significant associations are color-coded according to the legend (p values were based on two-sided Fisher’s exact test). Abbreviations: LA: lymphadenopathy

With specific regard to the CVID cohort, the 31 patients had a mean symptomatic disease duration of 13.7 years and exhibited a wide spectrum of clinical manifestations with patients displaying with one or multiple clinical features, as illustrated in Fig. 3. As shown in a direct comparison of frequency of clinical manifestations and relative odds ratios, we observed a clear association between the prevalence of splenomegaly, lymphadenopathy, and granulomatous manifestations. Indeed, the presence of granulomas strongly associated with splenomegaly and lymphadenopathy (LA), with an odds ratio of 12.9 for both conditions (p = 0.0217, Fisher’s exact test). Likewise, there was a significant correlation between the prevalence of LA and splenomegaly over the disease course (p = 0.0019, Fisher’s exact test).

Overall at the time of analysis, the vast majority of patients (94%) exhibited an infectious phenotype, primarily characterized by upper respiratory tract infections including otitis and sinusitis (61%), followed by lower respiratory infections (45%, with 50% of these cases diagnosed as pneumonia), gastrointestinal infections (31%), and a minority of skin infections (7%), urinary tract infections (7%), central nervous system infections (1 viral encephalitis), osteomyelitis, and sepsis (CMV, *C.jejuni*.) and 3 cases of recurrent cutaneous herpes zoster.

Among autoimmune and inflammatory manifestations observed in 19 patients (61%), autoimmune cytopenia was the most frequent disorder observed (29%). Other autoimmune diseases included connective tissue disorders (systemic sclerosis, rheumatoid arthritis, Sjogren syndrome, undifferentiated connective tissue disorders), gastrointestinal conditions (IBD-like, celiac-like disease, autoimmune atrophic gastritis), dermatological disorders (discoid lupus erythematosus, psoriasis, lichen planus), and Kawasaki disease.

Malignancies were diagnosed in 6 patients (19%) with B cell lymphoma being the most prevalent (2 patients). Other oncologic conditions were gastric cancer, thyroid carcinoma, testicular seminoma, and uterine carcinoma.

Six patients exhibited granulomatous diseases (lymph nodes, lungs, and spleen), 3 patients with multiple tissue localization.

**Fig. 4:**
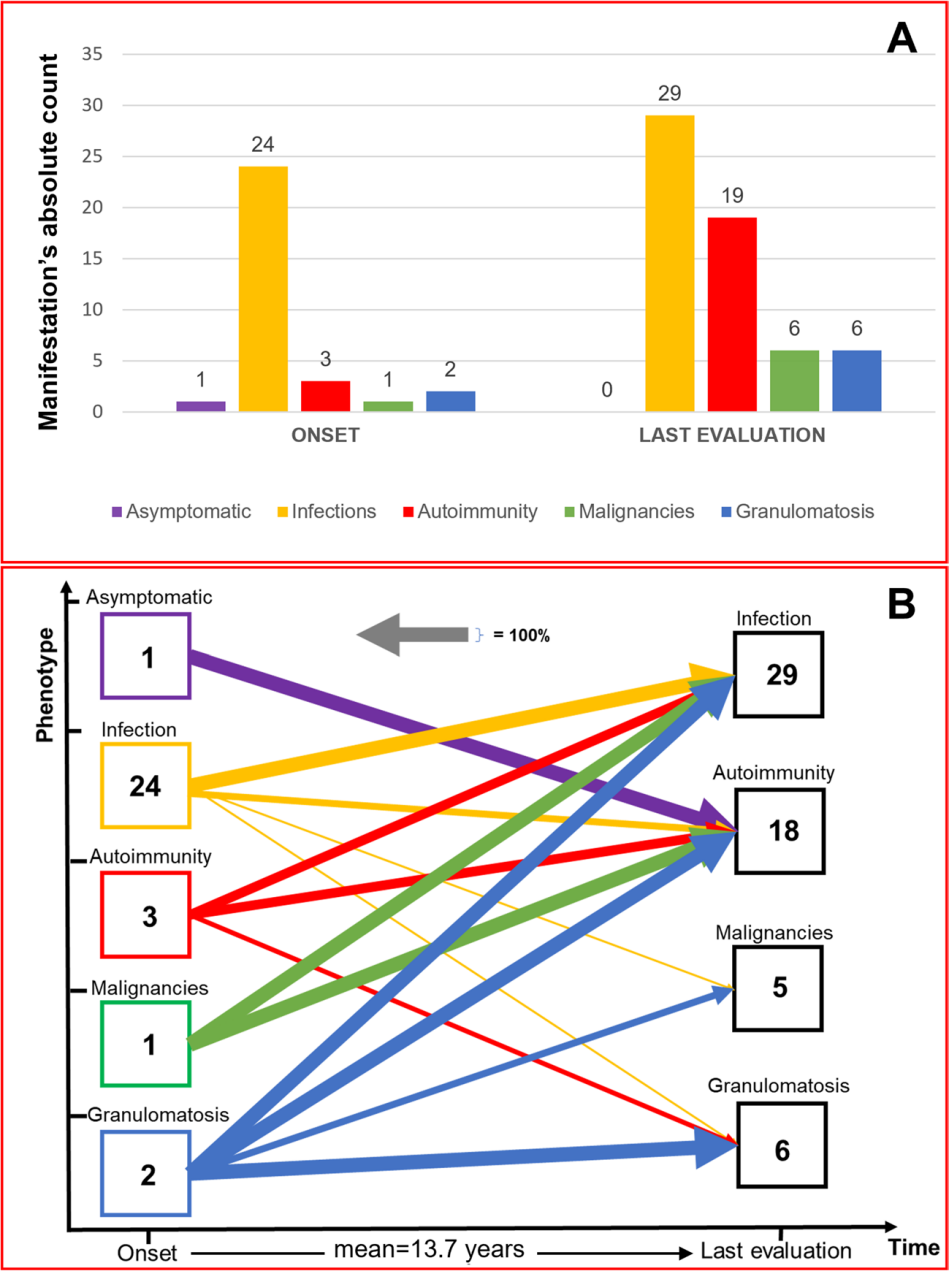
**A** Clinical phenotypes in CVID: onset vs. last clinical evaluation. Values represent the absolute patient count for each specific manifestation. **B** Evolution of clinical phenotypes over time. This graph illustrates the progression of clinical manifestations from onset to the last visit. “Onset” indicates syndrome which were present at first evaluation. “Last evaluation” indicates add-on syndromes to the one in “onset”. Onset manifestations, if limited on time or controlled by therapy (e.g., resolved neoplasm, autoimmune disease without further exacerbations, single infectious episode), are not considered among the manifestations at the last assessment. The thickness of each line indicates the percentage of patients who developed the condition indicated by the arrow, respect to the baseline (“onset”) number. For instance, the purple arrow for asymptomatic patients (1 individual) represents 100% because 1 out of 1 patient (hence, 100%) displayed an autoimmune phenotype during the last assessment. It is possible that the sum of the percentages of arrows of the same colour exceeds 100%, as the same patient may have presented different clinical manifestations. The numbers within the boxes represent the absolute number of patients who exhibited that specific manifestation in that specific time

In view of the prolonged observation and the apparent progressively occurring additional symptoms, we next studied the time course of new clinical disease manifestations. Our assessment evaluated symptoms at the initial presentation (onset of clinical symptoms) compared to those observed at the last visit (after an average of 13.7 years, IC95% 8.8–18.5 years) and showed that the infectious phenotype was the predominant initial manifestation (77% at onset vs. > 90% at the last clinical evaluation). Other clinical manifestations gradually increased over time, with autoimmune diseases showing a six-fold rise in prevalence (Fig. 4A).

To better understand dynamics of symptoms/syndromes over time in CVID patients, we elaborated data according to first presentation and subsequent syndromic evolutions for all patients and we drew the evolution of clinical phenotypes from the initial presentation to the final clinical assessment within the cohort (Fig. 4B). This analysis showed that, despite the onset, clinical manifestations have an exceptionally broad spectrum of evolution. Indeed, among the 24 patients who initially displayed an infectious phenotype, nearly 60% subsequently exhibited at least one additional clinical manifestation over time, including autoimmunity, malignancies, and granulomatous diseases. Notably, patients with an infectious onset and no additional complicating syndromes, had a significantly lower disease duration compared to those that developed autoimmunity, malignancies, or granulomatosis (means: 7.5 years [IC 95%, 1.1–13.9 years]; 18.43 years [IC95% 10.6–26.3 years], respectively; p = 0.0246, two-tailed Mann–Whitney U test).

Therefore, these observations challenge the routine clinical categorization of CVID patients based on specific phenotypes (e.g. complicated vs. non-complicated).

### CMV Viraemia is Detected in a Proportion of CVID Patients

As for CMV or EBV, viraemia with at least one of these two viruses was identified in 25% of the patients overall. CMV replication was observed in 16% of CVID and in 0% of non-CVID hID patients (p = 0.05, Fisher’s exact test). Conversely, EBV replication was equally distributed between the two populations. The mean CMV-DNA load was 380.9 IU/mL (range: 67–1382 IU/ml), while the mean EBV-DNA load was 2550.5 IU/mL (range:107–15288 IU/mL). No patients displayed clinical symptoms or laboratory abnormalities indicative of acute infection. CMV DNA detection in blood was not related to immunosuppressive medications. Specifically, among the CMV DNA-positive CVID patients, 4 out 5 were not treated with immunosuppressive drugs, while one patient was solely receiving low-dose of oral prednisone (5 mg/day) at the time of sampling.

Regarding EBV viraemia, 36% of the patients had either received or were currently undergoing immunosuppressive therapy. No differences were observed in terms of sex and age distribution between CMV and non-CMV patients (p = 0.637; p = 0.598, respectively), as well as between EBV and non-EBV patients (p = 0.318; p = 0.788, respectively). In addition, all patients tested negative in HHV6, HIV, HCV, and HBV RT-PCR blood testing.

Overall, patients with CMV viraemia had all a “complicated phenotype”, and a mean disease duration of 16.2 years, superimposable to the one of the “complicated phenotype” patients (18.4) years and clearly distinct from patients initially displaying a simple infectious clinical phenotype and no CMV viraemia (7.5 years). Further, CMV viraemic patients represented 24% of the patients with complicated phenotype (5 out of 21).

This suggests that, within a clinical evolution of CVID clinical phenotypes, CMV viraemia is present in only a subset of patients with complicated phenotype and higher disease duration.

### Reduced NK-cell Activation and Circulation of Inflammatory Precursors in CVID Patients with Active Viral Replication

Following the observation of virus circulation (EBV or CMV) in 26% of CVID patients, we addressed the question whether this lack of control could be associated with clinical and flow-cytometric phenotypes. To this end, we further studied the CVID cohort according to the presence/absence of viraemia (CMV only, EBV only, either CMV or EBV) (supplementary Tables S2-S4).

**Fig. 5:**
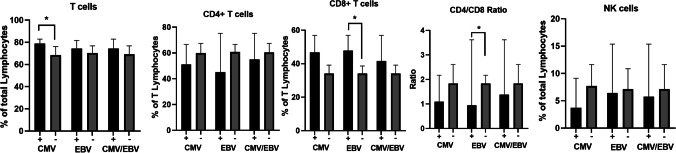
Comparison of lymphoid cell populations of CVID patients according to active ( +) or absent (-) viral replication of CMV-only, EBV only, and CMV and/or EBV (CMV/EBV) groups. Except for the CD4/CD8 ratio, all data is expressed as a percentage, specifically, T-cells and NK-cells as a percentage of total lymphocytes, while CD4 and CD8 as a percentage of T-cells. The histograms show the medians with CI 95%. P values are based on two-tailed Mann–Whitney U test (*p* < 0,05:*)

All these 3 groups were homogeneous in terms of demographic characteristics and disease duration. We observed, a strong association between CMV and EBV replication (OR = 11.5, p = 0.0376, Fisher’s exact test), with 3 patients displaying both CMV and EBV viraemia. Splenomegaly was the prevalent clinical finding in CMV and CMV/EBV patients (p = 0.0047 and p = 0.0316, respectively). No association was observed between immunoglobulin levels or B cell subpopulations and lack of viral control. There was a trend towards increased CD3^+^CD8^+^ T-cell frequencies in viraemic patients and reduced CD3^+^CD4^+^ T-cells, resulting in low CD4/CD8 ratio (Fig. 5).

**Fig. 6:**
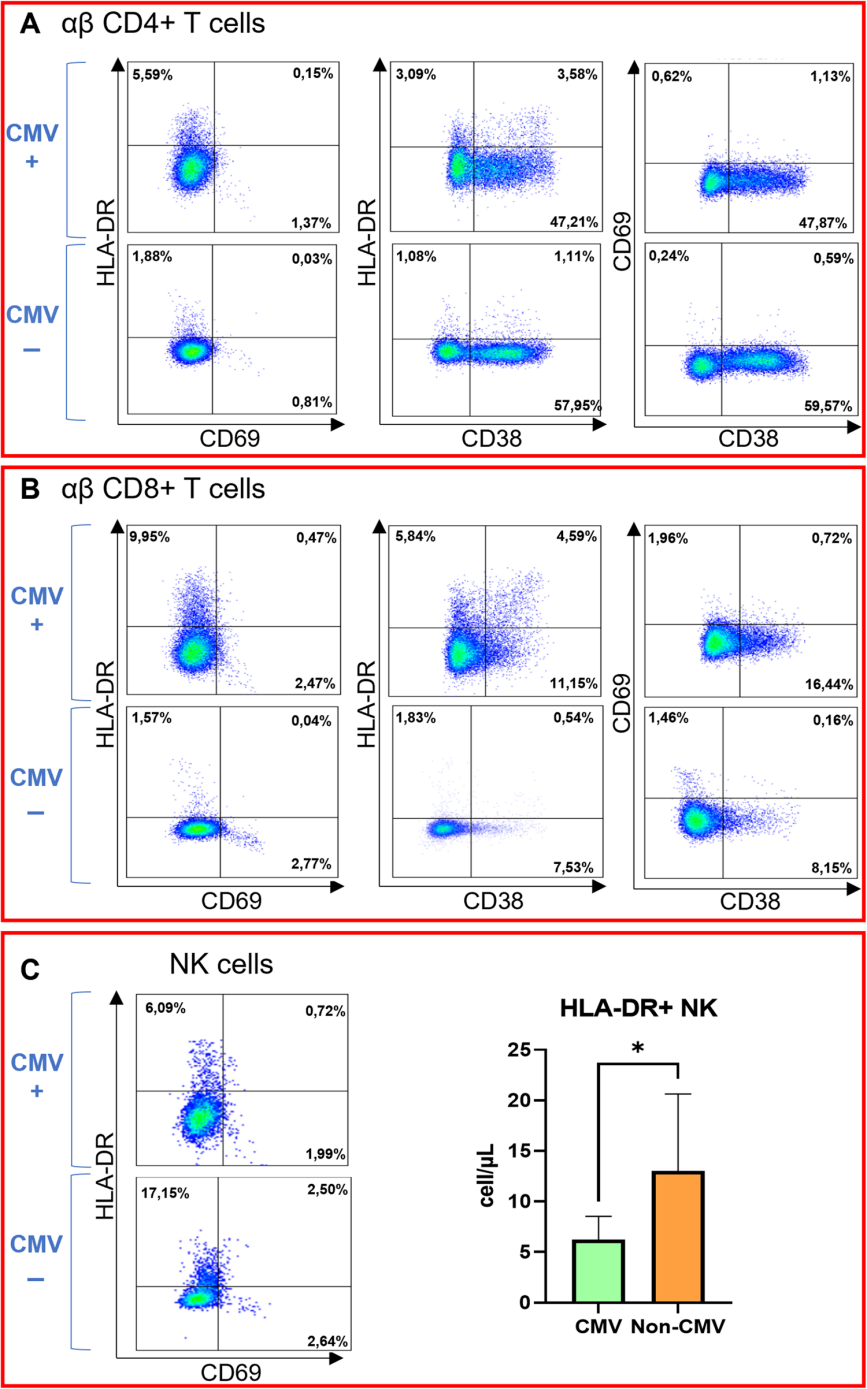
HLA-DR, CD38 and CD69 expression on T subsets and NK-cells. **A** Dot plots illustrate activation marker expression on CD4 + T-cells of both a CVID patient with CMV-only viraemia (CMV +) and a CVID patient without viraemia (CMV -). **B** Dot plots illustrate activation marker expression on CD8 + T-cells of both a CVID patient with CMV-only viraemia (CMV +) and a CVID patient without viraemia (CMV -). **C**. Dot plots illustrate HLA-DR and CD69 expression on NK-cells of both a CVID patient with CMV-only viraemia (CMV +) and a CVID patient without viraemia (CMV -). In the column bar graph, HLA-DR + NK-cell count between patients with active ( +) and absent (-) CMV replication was compared. The histograms represent the medians with CI 95%. P values are based on two-tailed Mann–Whitney U test (*p* < 0,05:*)

To investigate whether failure to control CMV viraemia might be associated with a dysregulation in cell activation, we further evaluated by flow cytometric analyses the expression of CD69, CD38 and HLA-DR molecules on T-cells and NK-cells (Fig. 6A and B). The expression of activation markers on CD4^+^ and CD8^+^ T-cells was similar in CVID viraemic and non-viraemic patients (supplementary Table S2). The mean absolute count of NK-cells was slightly decreased in all viraemic patients, with a trend towards a reduction in NK CD56^dim^ in the CMV group (p = 0.071). In CVID patients with CMV viraemia we observed a decrease in HLA-DR^+^ NK-cells (p = 0.048) without differences in CD69^+^HLA-DR^−^ and CD69^+^HLA-DR^+^ cell frequencies (Fig. 6B).

**Fig. 7:**
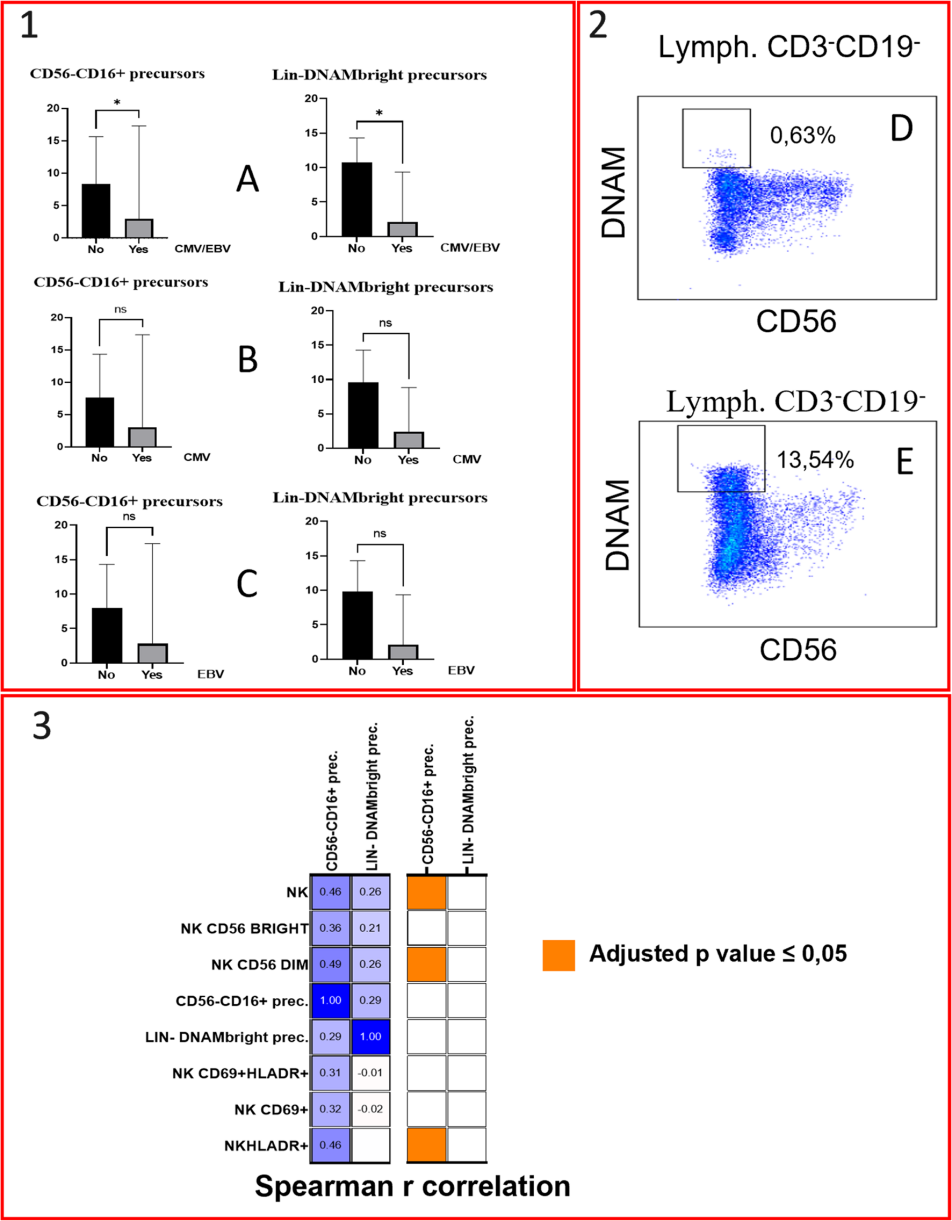
1. Comparison of lymphoid precursors (Lin-DNAMbright and Lin-CD16 +) absolute count between patients with active and absent viral replication. A: Either CMV or EBV replication; B: CMV-only; C: EBV-only. Histograms represent medians with CI 95%. P values are based on Mann–Whitney U test (ns = not significant; *p* < 0,05:*). 2. Dot plots illustrate LIN-DNAM^bright^ precursors of both a CVID patient with concurrent CMV and EBV viraemia (D) and a CVID patient without viraemia (E). 3. Correlation matrix of NK-cell subsets and lymphoid precursors absolute counts. The left figure depicts the correlation matrix, with each cell containing the Spearman r value between precursors and NK-cell subsets, while the right figure illustrates the adjusted p-values (by Benjamini–Hochberg correction) related to the same correlations using a color-coded legend. Abbreviations: Lin: lineage; Lymph: lymphocytes; Prec.: precursors

Finally, we analysed the circulation of inflammatory lymphoid precursors LIN^−^CD34^+^DNAM^bright^CXCR4^+^ and CD34^−^LIN^−^CD16^+^CD7^−^ precursors. Interestingly, the EBV/CMV viraemic group exhibited a reduction in both the absolute count of Lin^−^DNAM^bright^ and of CD56^−^CD16^+^ precursors (Fig. 7 and supplementary Table S4). Both CMV and EBV groups displayed a trend towards decrease in these cells. When considering other clinical outcomes such as autoimmunity, splenomegaly, granulomatous disease, LA, atopy, malignancies, and lung involvement, there were no significant differences in the absolute count of these cells compared to their respective aviremic control groups. The absolute count of CD56^−^CD16^+^ precursors positively correlated with the absolute number of NK-cells, NK CD56^dim^, and HLA-DR^+^ NK-cells (Fig. 7), in line with their ability to generate in vitro functional NK-cell progenies [29]. Conversely, DNAM^bright^ precursors did not exhibit any significant correlations with NK subsets. No correlations were identified between precursors and T-cell populations (total T-cells, CD4^+^, and CD8^+^ T-cells).

Taken together, these results show a possible involvement of NK-cells and inflammatory common lymphoid precursors in the lack of CMV control in a subset of CVID patients with prolonged disease course.

## Discussion

In the present study, we detected an impaired capacity to control CMV replication in a cohort of CVID patients with clinical and immunophenotypic features consistent with current clinical practice. This finding correlated with reduced numbers of inflammatory precursors and activated NK-cells. Additionally, we here found a temporal association between disease advancement and the gradual accumulation of symptoms, resulting in the development of what can be termed as "complicated" disease.

In view of the increasing number of scattered case reports [20, 21], the primary focus of the current study was to investigate CMV and EBV viraemia within a cohort of CVID patients and to explore their potential implications on clinical and pathophysiological aspects that have not been adequately addressed so far. Since patients described in single-case reports were often receiving immunosuppressive therapy predisposing to severe invasive CMV disease [38–40], the actual prevalence of lack of CMV control in CVID patients has been so far unexplored. Furthermore, although numerous cases of acute or disseminated CMV infection have been reported [20, 21], only one relatively old study specifically investigated CMV viraemia in CVID patients lacking acute CMV disease [41], reporting no apparent increase in frequency.

Here, we show that isolated CMV viraemia was a peculiarity of CVID patients (16%), suggesting that CMV-specific failure in immune control may represent a hallmark of a subset of CVID patients while it is not routinely observed in non-CVID hID.

Our data confirm for the first time that an immune dysregulation leading to a loss of CMV control, is present in 24% of complicated CVID patients, has no organ-specific invasive characteristics, and is usually neglected. These findings contribute to raising awareness of the impact of CMV on CVID disease within a specific patient subset, providing evidence for introducing CMV viremia as a beneficial screening tool for the assessment and management of these patients. In line with the knowledge that humoral immunity plays no/minor role in CMV/EBV replication, no significant differences were observed in the levels of immunoglobulin isotypes and B cell subtypes between viraemic and aviraemic patients. Conversely, several alterations in T and NK-cell subsets were observed, thus confirming that T-cell-mediated immunity plays the major role in suppressing viral replication. Indeed, host defense against CMV infection relies on both innate and adaptive cellular immune responses [42]. Early viral replication is primarily controlled by NK-cell cytokines and cytotoxicity [43, 44] and a critical role, in addition to CMV-specific T-cells, is played by NK-cells expressing high surface levels of CD94/NKG2C, which expand in CMV seropositive individuals [26, 45, 46].

Within our cohort, we observed a trend towards CD8^+^ T-cell expansion along with a CD4^+^ T-cell reduction leading to a reduced CD4/CD8 ratio in CVID patients with active viraemia vs. the negative group. Larger numbers of CVID viraemic patients are needed to verify this observation. Also, the distinct specific T-cell frequency and function reported to be present in blood compared to tissues compartments in donors with latent CMV infection can play a role [47].

Previously, an expansion of CMV-specific CD8^+^T-cells driven by CMV antigens in CMV tetramer-positive CVID patients has been linked to inflammatory complications [22]. There is however a relevant immune and virologic difference between the present report of CMV replication in PBMCs as compared to previous studies in patients with CMV latent infection without replication. Indeed, reports on CD3^+^ CD8^+^ late effector memory T cell expansion [22–24] reflect the ability of the immune system to control CMV, possibly leading to increased inflammaging. On the other hand, the present observation of failing control of CMV replication explores a different clinical condition compared to previous works, implying the existence of novel or additional defects. Notably, we here show for the first time, that in these CMV replicating patients, there is defect in NK cells and in inflammatory precursors which may represent one of the additional immune defects in these patients.

In fact, a significant reduction of circulating HLA-DR^+^ and a trend towards reduced CD56^dim^ NK-cells in CMV-only group was observed in this study, suggesting an impairment in NK-cell responses in CMV viraemic CVID patients. This is line with the recent observation that there is a significant reduction in NK-cell counts in CVID patients with disease complicated by non-infectious manifestations [34]. Further analysis of NKG2C^+^ adaptive NK-cells and of NK-cell function is therefore warranted to clarify CMV-specific function impairment in these patients.

Recently, the presence of two distinct peripheral blood precursors (CD34^+^DNAM^bright^CXCR4^+^ and Lin^−^CD56^−^CD16^+^Perf^−^CD94^−^CXCR4^+^ cells) has been described in patients with chronic infection including CMV, which originate in the bone marrow and migrate into the bloodstream [29, 30]. These precursors are able to rapidly differentiate into cytotoxic, IFNγ-secreting CD94/NKG2C^+^KIR^+^CD57^+^ CD56^dim^ NK-cell progenies that inhibit CMV replication [29]. Here, we observed a significant reduction in the absolute number of both these precursors in the CMV/EBV group vs. non-viraemic patients. Also, there was a direct correlation between CD56^−^CD16^+^ precursors (corresponding to CD34^−^CD3^−^CD19^−^CD56^−^CD16^+^CD7^−^CXCR4^+^ cells) and NK-cells subsets including total, CD56^dim^, and HLA-DR^+^ NK-cells. Notably, this defect in inflammatory precursors circulation was not evident when considering other clinical parameters of CVID patients (e.g. autoimmunity, splenomegaly, tumors, etc.). Taken together these findings indicate a bone marrow defect in producing or releasing these precursors into the bloodstream in CVID patients with CMV replication. This defect in precursors/stem cells would therefore be additive to other cell lineage precursor defects observed in CVID patients, as for B cells or T-cells. Future studies on CVID precursor differentiation in response to different stimuli and in different cytokine environments may provide a better understanding of the mechanism underlying this condition.

In this study we investigate the impact of CMV replication on CVID in a cross-sectional manner, exploring its associations with immunophenotypic and clinical profiles, and we do not have information on the duration of CMV replication in our CVID cohort. This prevents us from determining whether the observed deficiencies in CMV control are a late consequence of ongoing CMV replication (inflammation), or an early manifestation due to genetic predisposition in a subset of CVID patients. In case of a prolonged replication of CMV, this could indeed determine additional inflammaging in these patients and, at the same time, loss of CMV control late on the disease course could represent the consequence of inflammaging. Since however overt CMV replication is present in only 24% of complicated CVID patients, the possibility lies open of an original defect in CMV control early on at disease onset. Accordingly, the immunophenotypic alterations observed, could be either a result of chronic CMV exposure or a contributing factor to the impaired CMV control (or both), and need further evaluations. Overall, the present data warrant future change of patient management to regularly screen for CMV viraemia at diagnosis and to verify duration of CMV replication as well as its effect on inflammaging in these patients.

One of the main objectives of this work was to analyse clinical CVID phenotypes and their progression over time. So far, large multicentre studies have effectively delineated the clinical variability, and the correlations among different CVID clinical manifestations [2, 7, 17, 32]. There is however, a lack in a more comprehensive understanding of the clinical progression dynamic, starting from symptom onset through their accumulation over time.

One of the major concerns in clinical studies and in the literature is represented by the confusion between “clinical onset” and “symptoms at CVID diagnosis”. There is a distinct difference between “onset of clinical symptoms” and “symptoms at diagnosis”. Here, we represented data according to onset of clinical symptoms. The time lapse between onset of clinical symptoms and actual diagnostic procedures, may include several years. For these reasons, at diagnosis the patients often have more than one clinical manifestation of CVID, however, careful clinical assessment allowed to show that the majority of patients had infectious manifestations at “onset of clinical symptoms”.

Patients with characteristics of add-on non-infectious clinical pattern, are usually referred to be patients with “complicated disease”. We show here that the likelihood of developing a “complicated” phenotype is time-dependent, since patients with an infectious phenotype alone had a significantly shorter disease duration when compared to those who developed add-on non-infectious complications. This observation, has significant implications for patient management and for the design and interpretation of clinical studies, suggesting a careful re-evaluation of retrospective and cross-sectional clinical studies. Indeed, these studies have so far categorized CVID patient cohorts into distinct clinical phenotypes without considering disease duration [3, 31, 32, 34, 48]. Accordingly, some studies classify as "non-complicated disease" patients with only infectious manifestations of CVID of recent onset, who would subsequently develop a complex phenotype. Some studies report age of onset without reporting disease duration [3, 32, 34, 48] or even excluding from analysis patients with < 5 years of follow-up [6]. Based on present data, both cases introduce a considerable time-dependent bias in currently available clinical studies. Taken together therefore, the present data suggest that disease duration and evolution represent an essential parameter to follow-up CVID patients that needs to be considered in clinical and phenotypic studies.

The present study is subject to some limitations that are essential to consider when interpreting the results. Firstly, the power of our study is limited by its retrospective design, monocentric nature, and the relatively small sample size which precluded multivariate analysis. Another limitation is represented by the impossibility to assess patient exposure to CMV and EBV. Since patients had IgG deficiency and most of them were in Ig replacement therapy as expected in CVID clinical practice, serologic tests were not appropriate for this purpose. Since, however, our primary aim was to evaluate CVID patients with active viral replication vs, those without viraemia, the assumption of a 100% exposure status may in this case only have underestimated the correct prevalence of viral replication in the cohort.

## Conclusion

CVID patients have a crude prevalence of CMV viraemia of at least 16% in the absence of clinical manifestations of acute CMV infection/disease, nor progression to end-organ disease. Low numbers of circulating inflammatory lymphoid precursors and a reduction of activated HLA-DR^+^ NK-cells underlie the lack of control of CMV replication.

CMV replication in CVID patients is therefore more frequent than expected, and associates with prolonged disease duration and with immune imbalances to be considered in everyday patients’ clinical management.

Finally, the initial infectious phenotype indeed represents the most common clinical presentation at symptom onset but evolves in a time-dependent manner with add-on non-infectious clinical manifestations, leading to complicated phenotypes over time. Thus, complicated, and non-complicated disease do not represent disease severity per se but rather reflect disease duration and highlight the need for more critical attention to the design and interpretation of clinical studies in order to avoid potential selection bias.

### Supplementary Information

Below is the link to the electronic supplementary material.

Supplementary file1 (PDF 703 KB)

## Data Availability

The datasets generated and/or analysed during the current study are available from the corresponding author on reasonable request.
