# Supplementary material for: Evaluation of Frequency of CMV Replication and Disease Complications Reveals New Cellular Defects and a Time Dependent Pattern in CVID Patients
Source: J Clin Immunol. 2024 Jun 7;44(6):142. doi: 10.1007/s10875-024-01744-3 (PMC11161436; doi:10.1007/s10875-024-01744-3)
Supplement: Supplementary file 1 — Supplementary file1 (PDF 703 KB) [file 10875_2024_1744_MOESM1_ESM.pdf]

Evaluation of frequency of CMV replication and disease complications reveals new cellular defects and a time dependent pattern in CVID patients

Luca Marri[1,2], Paola Contini[1], Federico Ivaldi[1], Chiara Schiavi[2], Ottavia Magnani[2], Chiara Vassallo[2], Andrea Guastalla[2], Noemi Traversone[1,2], Claudia Angelini[3], Genny Del Zotto[4], Andrea De Maria[5,6,a], Raffaele De Palma[1,2,a]

a) Department of Internal Medicine (DIMI), University of Genoa, Genova, Italy.
b) Unit of Clinical Immunology and Translational Medicine, IRCCS Ospedale Policlinico San Martino, Genova, Italy.
c) Institute for Applied Mathematics "Mauro Picone", National Research Council, Napoli, Italy.
d) Integrated Department of Services and Laboratories, IRCCS Istituto Giannina Gaslini, Genova, Italy.
e) Department of Health Sciences (D.I.S.S.A.L.), University of Genoa, Genova, Italy
f) Infections in Immunocompromised Host Unit, IRCCS Ospedale Policlinico San Martino, Genova, Italy.

1. These authors contributed equally to this paper.

Address correspondence to:
Prof. Andrea De Maria, MD, Department of Health Sciences (D.I.S.S.A.L.), University of Genoa, Via A. Pastore, 1, 16132, Genova, Italy. E-mail: de-maria@unige.it
Prof. Raffaele De Palma, MD PhD, Department of Internal Medicine (DIMI), University of Genoa, Viale Benedetto XV, 6 16132, Genova, Italy. E-mail: raffaele.depalma@unige.it

Supplementary materials

Antibody panel composition

Anti-CD16 FITC, mouse IgG1. clone NKP15, BD Biosciences.

Anti-CD3 PerCP Cy5.5, mouse IgG1 k, clone SK7, BD Biosciences.

Anti-TCRγδ PE, mouse IgG1 k, clone B1, Biolegend.

Anti-CD69 PE-Cy7, mouse IgG1 k, clone FN50, BD Biosciences.

Anti-DNAM-1 (CD226) APC, mouse IgG1 k, clone 11A8, Biolegend.

Anti-CD38 AF700, mouse IgG1, clone LS198, Beckman Coulter.

Anti-CD8 APC-Cy7, mouse IgG1 k, clone SK1, Biolegend.

Anti-HLA-DR PB, mouse IgG2a k, clone L243, Biolegend.

Anti-CD45 KO, mouse IgG1, clone J33, Beckman Coulter.

Anti-CD19 BV605, mouse IgG1 k, clone SJ25C1, BD Biosciences.

Anti-CD56 BV650, mouse IgG1 k, clone 5.1H11, Biolegend.

Anti-CD4 BV786, mouse IgG2b k, clone OKT4, Biolegend.

Supplementary tables

Table S1. CVID V.S. non-CVID hID PATIENTS: IMMUNOLOGICAL VARIABLES					
	**TOTAL (52)**	**CVID (31)**	**NON-CVID (21)**	**MD**	**P value**
**IMMUNOGLOBULINS**					
IgG (onset) (g/L)	4.3 (3.5-5.1)	3.1 (2.3-4.0)	6.0 (4.6-7.4)	-2.88	**0.0003**
IgA (g/L)	0.55 (0.36-0.75)	0.48 (-0.02-0.18)	1.0 (0.6-1.4)	-0.53	**0.0002**
IgM (g/L)	0.42 (0.29-0.56)	0.38 (0.18-0.59)	0.48 (0.33-0.64)	-0.10	**0.0294**
IgE (kU/L)	19.0 (8.2-29.9)	10.1 (2.7-17.5)	39.1 (7.9-70.4)	-29	**0.0175**
**GENERAL IMMUNOPHENOTYPE (ABSOLUTE COUNT: CELLS/µL)**					
Lymphocytes	2045 (1765-2325)	2118 (1698-2538)	1933 (1597-2268)	185	0.782
Lymphocytes (% of WBC)	34.0 (31.1-36.9)	35.3 (31.2-39.5)	31.8 (28.1-35.6)	3.5	0.208
Monocytes	383 (333-434)	369 (306-432)	404 (315-493)	-35	0.554
Monocytes (% of WBC)	6.5 (5.8-7.1)	6.3 (5.3-7.2)	6.8 (5.9-7.7)	-0.5	0.169
T-cells	1357 (1167-1547)	1446 (1177-1716)	1225 (956-1494)	221	0.097
T-cells (% of Lymphocytes))	67.0 (63.8-70.3)	69.3 (65.4-73.2)	63.5 (57.7-69.4)	5.7	0.141
B cells	248 (170-326)	266 (155-378)	220 (108-332)	46	0.961
B cells (% of Lymphocytes)	11.1 (8.6-13.5)	10.9 (7.9-13.8)	11.4 (6.8-16.0)	-0.56	0.811
NK-cells	182 (144-221)	172 (121-222)	198 (135-261)	-26	0.175
NK-cells (% of Lymphocytes)	9.2 (7.5-10.8)	8.6 (6.5-10.7)	10.0 (7.0-10.7)	-1.4	0.364
NKT	97 (46-149)	58 (31-85)	156 (32-279)	-98	**0.027**
NKT-cells (% of Lymphocytes)	4.3 (2.2-6.5)	2.7 (1.8-3.6)	6.8 (1.5-12.1)	-4.1	0.080
Lineage negative (Lin-)	163 (137-188)	173 (134-211)	148 (118-179)	24	0.529
Lin- cells (% of lymphocytes)	8.8 (7.3-10.2)	9.4 (7.3-11.5)	7.8 (5.8-9.8)	1.6	0.459
**B CELLS IMMUNOPHENOTYPE (% OF TOTAL B CELLS)**					
Naïve $IgM^{+}IgD^{++}CD38^{+}CD27^{-}CD21^{+}$	50.4 (43.1-57.8)	54.4 (46.3-62.6)	39.2 (22.4-56.1)	15.2	0.0703
CD21low	8.9 (5.8-12.1)	9.4 (5.3-13.5)	7.7 (3.0-12.4)	1.7	0.888

$IgM^{+}IgD^{+}CD38^{low}CD27^{-}CD21^{low}$					
Class-switched memory $IgM^{-}IgD^{-}CD38^{+}CD27^{+}CD21^{+}$	5.3 (3.3-7.4)	4.1 (1.6-6.6)	8.4 (4.9-11.9)	-4.3	**0.0130**
Non-switched memory $IgM^{++}IgD^{+}CD38^{+}CD27^{+}CD21^{+}$	12.7 (7.5-17.8)	8.3 (4.1-12.5)	25.1 (9.9-40.2)	-16.8	**0.0087**
Transitional $IgM^{++}IgD^{++}CD38^{++}CD27^{-}CD21^{+}$	6.6 (3.2-9.9)	7.6 (3.2-12.0)	3.7 (-0.1-7.5)	3.9	0.104
Plasmablasts $IgM^{-/+}IgD^{-}CD38^{+++}CD27^{++}CD21^{+}$	0.34 (0.09-0.60)	0.37 (0.05-0.69)	0.27 (-0.18-0.73)	0.10	0.962
**T-CELLS IMMUNOPHENOTYPE**					
αβ T-cells	1327 (1140-1515)	1409 (1145-1673)	1206 (935-1477)	203	0.113
αβ **T-cells (% of T-cells)**	97.7 (97.1-98.2)	97.4 (96.8-98.1)	98.0 (97.0-99.1)	-0.6	**0.020**
γδ T-cells	29.8 (21.7-37.9)	37.1 (24.8-49.4)	19.0 (11.4-26.5)	18.1	**0.004**
γδ **T-cells (% of T-cells)**	2.3 (1.8-2.8)	2.6 (1.9-3.2)	1.9 (0.9-3.0)	0.6	**0.019**
αβ CD4+ T	808 (697-918)	805 (667-942)	812 (614-1010)	-8	0.671
αβ CD4+ T (% of αβ T-cells)	61.9 (58.8-65.1)	58.7 (54.4-63.2)	66.6 (62.8-70.3)	-7.8	**0.012**
αβ CD8+ T	455 (360-550)	528 (384-672)	341 (258-425)	187	**0.045**
αβ CD8+ T (% of αβ T-cells)	33.0 (30.1-35.9)	35.7 (31.6-39.7)	28.8 (25.2-32.4)	6.9	**0.016**
αβ DN T-cells	59.3 (46.1-72.6)	68.0 (48.5-87.4)	46.0 (30.6-61.4)	22.0	0.095
αβ **DN T-cells (% of** αβ **T-cells)**	4.6 (3.8-5.4)	5.0 (3.9-6.1)	4.0 (2.7-5.3)	1.0	0.163
αβ CD4+CD8+ T	9.3 (4.9-13.6)	8.5 (2.6-14.3)	10.5 (3.5-17.6)	-2.0	0.931
αβ CD4+CD8+ T (% of αβ T-cells)	0.67 (0.40-0.94)	0.52 (0.24-0.80)	0.9 (0.3-1.5)	-0.38	0.871
**NK IMMUNOPHENOTYPE (% OF TOTAL NK-CELLS)**					
NK $CD56^{dim}$	168.0 (131.0-205.0)	157.3 (108.4-206.2)	168.4 (112.4-224.5)	-26.4	0.169
NK $CD56^{dim}$ %	90.8 (87.9-93.6)	90.6 (86.4-94.8)	91.0 (87.0-95.1)	-0.45	0.345
NK $CD56^{bright}$	11.4 (9.1-13.7)	10.3 (7.8-12.8)	13.1 (8.5-17.6)	-2.8	0.335
NK $CD56^{bright}$ %	7.7 (6.3-9.0)	7.31 (6.11-8.51)	8.2 (5.2-11.2)	-0.86	0.505

Quantitative variables are expressed as means with a 95% confidence interval, while qualitative variables are represented as absolute frequencies with percentages relative to the total of the respective column. P values were calculated by either unpaired two-tailed Student's t-test or Mann-Whitney u test according to variable distribution and sample size. OR: odds ratio; MD: mean difference; DN: double negative

Table S2. **Comparison between CVID patients with active CMV replication vs non-replicating**

	Total (31)	CMV (5)	Non/CMV (26)	OR/ MD	P value (univar.)
**SAMPLE DEMOGRAPHICS**					
Male	14(45%)	1(20%)	13(50%)	0.25	0.3445
Female	17(55%)	4(80%)	13(50%)	4.0	0.3445
Age	50.4 (44.1-56.7)	56 (37.0-75.0)	49.4 (42.3-56.4)	6.7	0.4352
**CLINICAL VARIABLES**					
Age at diagnosis	39.0 (32.3-45.8)	41.8 (18.8-64.8)	38.5 (30.9-46.1)	3.3	0.6861
Disease duration	13.7 (8.8-18.5)	16.2 (3.6-28.8)	13.2 (7.6-18.7)	3.0	0.4078
Ig replacement therapy (IGRT)	23(74%)	4(80%)	19(73%)	1.474	>0.9999
IGRT duration	8.1 (4.8-11.4)	12.0 (1.7-22.3)	7.2 (3.6-10.8)	4.8	0.3112
Infections	29(94%)	5(100%)	24(92%)	Infinity	>0.9999
Autoimmunity	19(61%)	3(60%)	16(62%)	0.9375	>0.9999
Splenomegaly	12(39%)	5(100%)	7(27%)	Infinity	**0.0047**
Granulomatosis	6(19%)	2(40%)	4(15%)	3.667	0.2020
Malignancies	6(19%)	1(20%)	5(19%)	1.05	0.9682
Lymphadenomegaly	12(39%)	4(80%)	8(31%)	9.0	0.0600
Lung involvement	13(42%)	4(80%)	9(35%)	7.556	0.1337
Atopy	10(32%)	0(0%)	10(38%)	0	0.1472
Blood EBV replication	6(19%)	3(60%)	3(12%)	11.5	**0.0376**
**IMMUNOGLOBULINS**					
IgG (onset) (g/L)	3.1 (2.3-4.0)	1.6 (-1.1-4.4)	3.3 (2.4-4.2)	-1.7	0.2740
IgA (g/L)	0.48 (-0.02-0.18)	0.11 (-0.16-0.38)	0.56 (-0.04-1.15)	-0.45	0.2498
IgM (g/L)	0.38 (0.18-0.59)	0.35 (-0.25-0.95)	0.39 (0.15-0.62)	-0.04	0.8870
IgE (kU/L)	10.1 (2.7-17.5)	2.2 (-4.0-8.5)	11.9 (2.9-20.9)	-9.7	0.3323
**GENERAL IMMUNOPHENOTYPE (ABSOLUTE COUNT: CELLS/µL)**					
Lymphocytes	2118 (1698-2538)	2220 (157-4283)	2098 (1669-2528)	121	0.584
Lymphocytes (% of WBC)	35.3 (31.2-39.5)	38.1 (17.9-58.2)	34.9 (30.6-39.1)	3.2	0.480
Monocytes	369 (306-432)	376 (55-698)	368 (305-431)	9	0.696
Monocytes (% of WBC)	6.3 (5.3-7.2)	6.0 (4.4-7.5)	6.3 (5.1-7.5)	-0.4	1.000
T cells	1446 (1177-1716)	1675 (226-3124)	1402 (1141-1664)	273	0.897
T cells (% of Lymphocytes)	69.3 (65.4-73.2)	77.3 (70.6-84.1)	67.7 (63.4-72.1)	9.6	**0.041**
B cells	266 (155-378)	252 (-177-681)	261 (142-380)	-9	0.514
B cells (% of Lymphocytes)	10.9 (7.9-13.8)	8.3 (1.6-14.9)	11.4 (8.0-14.7)	-3.1	0.548
NK cells	172 (121-222)	97 (23-170)	186 (127-245)	-90	0.235
NK cells (% of Lymphocytes)	8.6 (6.5-10.7)	5.2 (1.3-9.1)	9.3 (6.9-11.6)	-4.0	0.159
NKT-LIKE	58 (31-85)	87 (-74-249)	52 (28-76)	35	0.735
NKT-LIKE cells (% of Lymphocytes)	2.7 (1.8-3.6)	2.7 (-0.6-6.1)	2.7 (1.7-3.7)	0.06	0.856
Lineage negative (Lin-)	173 (134-211)	129 (36-221)	181 (137-225)	-52	0.358
Lin- cells (% of lymphocytes)	9.4 (7.3-11.5)	7.8 (1.8-13.8)	9.7 (7.4-12.1)	-1.9	0.658

**B CELLS IMMUNOPHENOTYPE (% OF TOTAL B CELLS)**					
Naïve<br>IgM^{+}IgD^{++}CD38^{+}CD27^{-}CD21^{+}	54.4<br>(46.3-62.6)	58.5<br>(39.3-77.7)	53.7<br>(44.1-63.2)	4.8	0.8987
CD21low<br>IgM^{+}IgD^{+}CD38^{low}CD27^{-}CD21^{low}	9.4<br>(5.3-13.5)	15.4<br>(-5.0-35.7)	8.2<br>(4.2-12.3)	7.2	0.1724
Class-switched memory<br>IgM^{-}IgD^{-}CD38^{+}CD27^{+}CD21^{+}	4.1<br>(1.6-6.6)	2.3<br>(-0.1-4.8)	4.5<br>(1.5-7.4)	-2.2	0.9688
Non-switched memory<br>IgM^{++}IgD^{+}CD38^{+}CD27^{+}CD21^{+}	8.3<br>(4.1-12.5)	3.6<br>(1.7-5.4)	9.1<br>(4.2-14.1)	-5.5	0.8450
Transitional<br>IgM^{++}IgD^{++}CD38^{++}CD27^{-}CD21^{+}	7.6<br>(3.2-12.0)	8.2<br>(0.04-16.4)	7.5<br>(2.2-12.7)	0.7	0.2448
Plasmablasts<br>IgM$^{-/+}$IgD^{-}CD38^{+++}CD27^{++}CD21^{+}	0.37<br>(0.05-0.69)	0.78<br>(-1.18-2.74)	0.29<br>(0.01-0.56)	0.49	0.5366
**T CELLS IMMUNOPHENOTYPE**					
αβ T cells	1409<br>(1145-1673)	1644<br>(197-3092)	1364<br>(1112-1616)	280	0.938
αβ T cells<br>(% of T cells)	97.4<br>(96.8-98.1)	97.9<br>(95.4-100.4)	97.4<br>(96.7-98.0)	0.6	0.176
γδ T cells	37.1<br>(24.8-49.4)	30.5<br>(-8.2-69.2)	38.4<br>(24.5-52.3)	-7.9	0.480
γδ T cells<br>(% of T cells)	2.6<br>(1.9-3.2)	2.1<br>(-0.5-4.6)	2.6<br>(2.0-3.3)	-0.6	0.176
αβ CD4+ T	805<br>(667-942)	770<br>(306-1233)	811<br>(657-966)	-42	0.775
αβ CD4+ T<br>(% of αβ T cells)	58.7<br>(54.4-63.2)	50.8<br>(34.4-67.2)	60.3<br>(55.7-65.0)	-9.5	0.195
αβ CD8+ T	528<br>(384-672)	787<br>(-139-1713)	478<br>(364-593)	309	0.417
αβ CD8+ T<br>(% of αβ T cells)	35.7<br>(31.6-39.7)	44.0<br>(31.0-56.9)	34.1<br>(29.8-38.3)	9.9	0.081
αβ DN T cells	68.0<br>(48.5-87.4)	68.3<br>(-6.8-143.4)	67.9<br>(46.6-89.2)	0.5	0.775
αβ DN T cells<br>(% of αβ T cells)	5.0<br>(3.9-6.1)	4.5<br>(-0.5-9.6)	5.1<br>(4.0-6.2)	-0.6	0.257
αβ CD4+CD8+ T	8.5<br>(2.6-14.3)	19.5<br>(-25.0-64.1)	6.4<br>(3.0-9.8)	13.1	0.979
αβ CD4+CD8+ T<br>(% of αβ T cells)	0.52<br>(0.24-0.80)	0.70<br>(-0.40-1.80)	0.49<br>(0.18-0.79)	0.21	0.735
αβ CD4+DR+ T	36.1<br>(27.6-44.5)	35.3<br>(4.1-66.5)	36.2<br>(26.9-45.6)	-0.9	0.815
αβ CD4+DR+ T<br>(% of CD4 αβ T cells)	5.1<br>(3.9-6.2)	5.6<br>(0.7-10.5)	5.0<br>(3.8-6.2)	0.6	0.815
αβ CD4+CD69+ T	11.6<br>(8.2-15.1)	11.2<br>(1.4-21.0)	11.7<br>(7.7-15.7)	-0.5	0.938
αβ CD4+CD69+ T<br>(% of CD4 αβ T cells)	1.6<br>(1.0-2.1)	1.5<br>(0.3-2.8)	1.6<br>(0.9-2.2)	-0.02	0.696
αβ CD4+DR+CD69+ T	1.1<br>(0.6-1.6)	1.6<br>(-1.3-4.6)	1.0<br>(0.6-1.4)	0.6	0.938
αβ CD4+DR+CD69+ T<br>% of CD4 αβ T cells	0.2<br>(0.1-0.2	0.2<br>(-0.2-0.6)	0.1<br>(0.07-0.2)	0.1	0.658
αβ CD4+DR+CD38+ T	13.8<br>(8.9-18.8)	23.7<br>(2.5-45.0)	11.9<br>(7.0-16.9)	11.8	0.063
αβ CD4+DR+CD38+ T<br>(% of CD4 αβ T cells)	2.1<br>(1.4-2.8)	3.7<br>(0.6-6.8)	1.7<br>(1.1-2.4)	2.0	0.103
αβ CD4+CD38+CD69+ T	4.6<br>(3.0-6.3)	4.0<br>(1.8-6.2)	4.7<br>(2.8-6.7)	-0.7	0.620
αβ CD4+CD38+CD69+ T<br>(% of CD4 αβ T cells)	0.6<br>(0.4-0.8)	0.6<br>(0.2-1.0)	0.6<br>(0.4-0.8)	-0.01	0.897
αβ CD8+DR+ T	32.2<br>(22.1-42.3)	40.1<br>(15.8-64.4)	30.7<br>(19.0-42.3)	9.5	0.214
αβ CD8+DR+ T<br>(% of CD8 αβ T cells)	6.9<br>(5.3-8.4)	7.8<br>(0.9-14.7)	6.7<br>(5.1-8.3)	1.1	0.735
αβ CD8+CD69+ T	22.1<br>(6.7-37.6)	28.6<br>(-8.7-65.9)	20.9<br>(2.9-38.8)	7.7	0.235
αβ CD8+CD69+ T<br>(% of CD8 αβ T cells)	4.3<br>(1.3-7.3)	3.4<br>(2.9-3.9)	4.4<br>(0.8-8.1)	-1.0	0.115
αβ CD8+DR+CD69+ T	1.6	2.6	1.4	1.2	0.214

	(1.1-2.1)	(-0.3-5.5)	(1.0-1.9)		
αβ CD8+DR+CD69+ T (% of CD8 αβ T cells)	0.4 (0.3-0.5)	0.5 (0.03-0.9)	0.3 (0.2-0.5)	0.1	0.448
αβ CD8+DR+CD38+ T	13.5 (8.2-18.8)	21.3 (4.8-37.9)	12.0 (6.1-17.9)	9.3	0.055
αβ CD8+DR+CD38+ T (% of CD8 αβ T cells)	2.7 (1.9-3.4)	4.0 (0.3-7.7)	2.4 (1.7-3.1)	1.6	0.214
αβ CD8+CD38+CD69+ T	3.8 (1.5-6.1)	9.4 (-7.0-25.8)	2.7 (1.4-4.0)	6.7	0.103
αβ CD8+CD38+CD69+ (% of CD8 αβ T cells)	0.7 (0.4-0.9)	0.9 (0.3-1.5)	0.6 (0.3-0.9)	0.3	0.115
**NK IMMUNOPHENOTYPE (% OF TOTAL NK CELLS)**					
NK CD56^{dim}	157.3 (108.4-206.2)	66.3 (34.7-97.9)	174.8 (118.8-230.9)	-108.6	0.071
NK CD56^{dim} %	90.6 (86.4-94.8)	80.3 (46.3-114.3)	92.6 (91.2-93.9)	-12.2	0.280
NK CD56^{bright}	10.3 (7.8-12.8)	5.6 (3.4-7.8)	11.2 (8.3-14.1)	-5.5	0.103
NK CD56^{bright} %	7.31 (6.11-8.51)	6.84 (4.16-9.51)	7.39 (6.03-8.76)	-0.59	0.979
NK DR+	16.7 (11.8-21.5)	7.0 (4.3-9.7)	18.5 (13.0-24.1)	-11.6	**0.048**
NK DR+ %	13.2 (8.8-17.6)	9.3 (3.6-15.1)	13.9 (8.7-19.2)	-4.6	0.735
NK CD69+	9.5 (4.8-14.1)	3.3 (1.2-5.4)	10.7 (5.2-16.2)	-7.3	0.387
NK CD69+ %	6.7 (3.4-10.0)	4.2 (0.6-7.9)	7.2 (3.3-11.1)	-3.0	0.735
NK DR+CD69+	2.7 (-0.2-5.5)	0.5 (-0.03-1.0)	3.1 (-0.3-6.5)	-2.6	0.448
NK DR+CD69+ %	2.8 (-0.3-5.8)	0.5 (0.2-0.9)	3.2 (-0.4-6.9)	-2.7	0.696
**PUTATIVE LYMPHOID PRECURSORS**					
PUTATIVE CD56-CD16+ PRECURSORS	11.3 (7.5-15.1)	5.94 (-3.16-15.05)	12.34 (8.04-16.64)	-6.40	0.129
PUTATIVE CD56-CD16+ PRECURSORS%	3.45 (2.23-4.67)	3.61 (-2.98-10.20)	3.42 (2.23-4.61)	0.19	0.514
LIN-DNAM^{bright}	9.99 (6.56-13.41)	3.74 (-0.46-7.93)	11.19 (7.28-15.09)	-7.45	0.195
LIN-DNAM^{bright} %	3.06 (1.85-4.27)	1.60 (0.41-2.78)	3.34 (1.92-4.77)	-1.75	0.514

Quantitative variables were expressed as means with a 95% confidence interval, while qualitative variables were represented as absolute frequencies with percentages relative to the total of the respective column. P values were calculated by either unpaired two-tailed Student's t-test or Mann-Whitney u test according to variable distribution and sample size. OR: odds ratio; MD: mean difference; DN: double negative; Univar.: univariate analysis.

Table S3. Comparison between CVID patients with active EBV replication vs non-replicating

	Total (31)	EBV (6)	Non-EBV (25)	OR/ MD	P value (univar.)
**SAMPLE DEMOGRAPHICS**					
Male	14(45%)	3(50%)	11(44%)	1.273	>0.9999
Female	17(55%)	3(50%)	14(56%)	0.7857	>0.9999
Age	50.4 (44.1-56.7)	49.8 (30.0-69.7)	50.6 (43.5-57.6)	-0.7	0.9274
**CLINICAL VARIABLES**					
Age at diagnosis	39.0 (32.3-45.8)	37.5 (16.2-58.8)	39.4 (31.8-47.0)	-1.9	0.9901
Disease duration	13.7 (8.8-18.5)	13.7 (2.5-24.9)	13.6 (7.9-19.3)	0.03	0.7231
Ig replacement therapy (IGRT)	23(74%)	4(67%)	19(76%)	0.6316	0.6344
IGRT duration	8.1 (4.8-11.4)	8.0 (-3.4-19.4)	8.1 (4.5-11.8)	-0.1	0.9863
Infections	29(94%)	6(100%)	23(92%)	Infinity	>0.9999
Autoimmunity	19(61%)	3(50%)	16(64%)	0.5625	0.6526
Splenomegaly	12(39%)	4(67%)	8(32%)	4.25	0.1735
Granulomatosis	6(19%)	0(0%)	6(24%)	0	0.1815
Malignancies	6(19%)	2(33%)	4(16%)	2.625	0.3345
Lymphadenomegaly	12(39%)	3(50%)	9(36%)	1.778	0.6526
Lung involvement	13(42%)	3(50%)	10(40%)	1.500	0.6758
Atopy	10(32%)	0(0%)	10(40%)	0	0.1411
Blood EBV replication	6(19%)	3(50%)	2(8%)	11.5	**0.0376**
**IMMUNOGLOBULINS**					
IgG (onset) (g/L)	3.1 (2.3-4.0)	3.6 (1.7-5.5)	3.1 (2.1-4.0)	0.5	0.6446
IgA (g/L)	0.48 (-0.02-0.18)	0.35 (0.01-0.69)	0.52 (-0.11-1.14)	-0.17	0.2023
IgM (g/L)	0.38 (0.18-0.59)	0.36 (-0.07-0.80)	0.39 (0.14-0.63)	-0.03	0.8171
IgE (kU/L)	10.1 (2.7-17.5)	8.7 (-8.6-26.0)	10.5 (1.6-19.5)	-1.8	0.7787
**GENERAL IMMUNOPHENOTYPE (ABSOLUTE COUNT: CELLS/ µL)**					
Lymphocytes	2118 (1698-2538)	2262 (683-3841)	2084 (1638-2529)	178	0.865
Lymphocytes (% of WBC)	35.3 (31.2-39.5)	35.6 (20.1-51.0)	35.3 (30.9-39.8)	0.2	0.789
Monocytes	369 (306-432)	426 (205-646)	356 (288-423)	70	0.575
Monocytes (% of WBC)	6.3 (5.3-7.2)	6.5 (5.4-7.7)	6.2 (5.0-7.4)	0.3	0.227
T cells	1446 (1177-1716)	1663 (510-2815)	1395 (1130-1659)	268	0.715
T Lymphocytes (% of Lymphocytes)	69.3 (65.4-73.2)	73.3 (65.9-80.8)	68.3 (63.6-73.0)	5.0	0.339
B cells	266 (155-378)	243 (-81-566)	263 (139-387)	-21	0.827
B cells (% of Lymphocytes)	10.9 (7.9-13.8)	9.0 (3.3-14.6)	11.3 (7.8-14.8)	-2.4	0.643
NK cells	172 (121-222)	145 (55-235)	178 (117-240)	-34	0.942
NK cells (% of Lymphocytes)	8.6 (6.5-10.7)	7.8 (2.6-13.0)	8.8 (6.4-11.2)	-1.0	0.751
NKT-LIKE	58 (31-85)	77 (-44-199)	53 (28-79)	24	0.827
NKT-LIKE cells (% of Lymphocytes)	2.7 (1.8-3.6)	2.6 (0.4-4.7)	2.7 (1.6-3.8)	-0.2	0.865
Lineage negative (Lin-)	173 (134-211)	176 (63-288)	172 (128-215)	4	0.935
Lin- cells (% of lymphocytes)	9.4 (7.3-11.5)	9.8 (4.2-15.3)	9.3 (6.9-11.7)	0.4	0.715

**B CELLS IMMUNOPHENOTYPE (% OF TOTAL B CELLS)**					
Naïve IgM^{+}IgD^{++}CD38^{+}CD27^{-}CD21^{+}	54.4 (46.3-62.6)	61.3 (46.2-66.3)	53.1 (43.6-62.7)	-8.2	0.5540
CD21low IgM^{+}IgD^{+}CD38^{low}CD27^{-}CD21^{low}	9.4 (5.3-13.5)	11.2 (-6.2-28.6)	8.9 (4.8-13.1)	-2.3	0.9133
Class-switched memory IgM^{-}IgD^{-}CD38^{+}CD27^{+}CD21^{+}	4.1 (1.6-6.6)	1.5 (0.2-2.8)	4.7 (1.7-7.8)	3.2	0.5995
Non-switched memory IgM^{++}IgD^{+}CD38^{+}CD27^{+}CD21^{+}	8.3 (4.1-12.5)	3.3 (1.3-5.3)	9.5 (9.3-14.6)	6.2	0.5168
Transitional IgM^{++}IgD^{++}CD38^{++}CD27^{-}CD21^{+}	7.6 (3.2-12.0)	6.2 (4.0-8.4)	7.9 (2.3-13.5)	1.7	0.1459
Plasmablasts IgM$^{-/+}$IgD^{-}CD38^{+++}CD27^{++}CD21^{+}	0.37 (0.05-0.69)	0.87 (-0.61-2.35)	0.25 (-0.03-0.52)	-0.63	0.0627
**T CELLS IMMUNOPHENOTYPE**					
αβ T cells	1409 (1145-1673)	1631 (480-2782)	1356 (1101-1611)	275	0.751
αβ T cells (% of T cells)	97.4 (96.8-98.1)	97.8 (95.9-99.7)	97.4 (96.7-98.1)	0.5	0.339
γδ T cells	37.1 (24.8-49.4)	31.8 (2.6-61.1)	38.4 (23.9-52.9)	-6.5	0.715
γδ T cells (% of T cells)	2.6 (1.9-3.2)	2.2 (0.3-4.1)	2.6 (1.9-3.3)	-0.4	0.364
αβ CD4+ T	805 (667-942)	743 (323-1164)	819 (664-975)	-76	0.608
αβ CD4+ T (% of αβ T cells)	58.7 (54.4-63.2)	49.4 (33.0-65.8)	61.1 (56.8-65.3)	-11.7	0.067
αβ CD8+ T	528 (384-672)	781 (66-1497)	467 (353-582)	314	0.247
αβ CD8+ T (% of αβ T cells)	35.7 (31.6-39.7)	43.9 (29.6-58.3)	33.7 (29.8-37.6)	10.3	0.053
αβ DN T cells	68.0 (48.5-87.4)	89.0 (33.6-144.5)	62.9 (41.1-84.7)	26	0.268
αβ DN T cells (% of αβ T cells)	5.0 (3.9-6.1)	6.1 (2.3-9.8)	4.8 (3.6-5.9)	1.3	0.364
αβ CD4+CD8+ T	8.5 (2.6-14.3)	17.0 (-17.3-51.3)	6.4 (2.9-10.0)	10.6	0.903
αβ CD4+CD8+ T (% of αβ T cells)	0.52 (0.24-0.80)	0.62 (-0.22-1.47)	0.50 (0.18-0.81)	0.12	0.679
αβ CD4+DR+ T	36.1 (27.6-44.5)	32.8 (11.4-54.1)	36.9 (27.0-46.8)	-4.1	0.865
αβ CD4+DR+ T (% of CD4 αβ T cells)	5.1 (3.9-6.2)	5.3 (2.0-8.6)	5.0 (3.7-6.3)	0.3	0.751
αβ CD4+CD69+ T	11.6 (8.2-15.1)	10.4 (2.2-18.6)	11.9 (7.9-16.0)	-1.6	0.942
αβ CD4+CD69+ T (% of CD4 αβ T cells)	1.6 (1.0-2.1)	1.7 (0.5-2.8)	1.5 (0.9-2.2)	0.1	0.478
αβ CD4+DR+CD69+ T	1.1 (0.6-1.6)	1.5 (-0.8-3.8)	1.0 (0.5-1.4)	0.5	0.827
αβ CD4+DR+CD69+ T % of CD4 αβ T cells	0.2 (0.1-0.2	0.2 (0.1-0.5)	0.1 (0.07-0.2)	0.1	0.339
αβ CD4+DR+CD38+ T	13.8 (8.9-18.8)	16.7 (-0.4-33.8)	13.1 (7.7-18.5)	3.6	0.510
αβ CD4+DR+CD38+ T (% of CD4 αβ T cells)	2.1 (1.4-2.8)	2.8 (0.2-5.4)	1.9 (1.1-2.6)	0.9	0.643
αβ CD4+CD38+CD69+ T	4.6 (3.0-6.3)	5.7 (0.2-11.2)	4.4 (2.6-6.1)	1.4	0.364
αβ CD4+CD38+CD69+ T (% of CD4 αβ T cells)	0.6 (0.4-0.8)	0.8 (0.3-1.3)	0.6 (0.4-0.7)	0.2	0.247
αβ CD8+DR+ T	32.2 (22.1-42.3)	39.9 (21.5-58.3)	30.3 (18.2-42.5)	9.5	0.105
αβ CD8+DR+ T (% of CD8 αβ T cells)	6.9 (5.3-8.4)	7.6 (3.5-11.8)	6.7 (4.9-8.5)	0.9	0.510
αβ CD8+CD69+ T	22.1 (6.7-37.6)	26.1 (-3.2-55.4)	21.2 (2.5-39.9)	4.9	0.247
αβ CD8+CD69+ T (% of CD8 αβ T cells)	4.3 (1.3-7.3)	3.2 (2.3-4.2)	4.5 (0.8-8.3)	-1.3	0.227
αβ CD8+DR+CD69+ T	1.6	2.4	1.4	0.9	0.208

	(1.1-2.1)	(0.2-4.6)	(0.9-1.9)		
αβ CD8+DR+CD69+ T (% of CD8 αβ T cells)	0.4 (0.3-0.5)	0.5 (0.1-1.0)	0.3 (0.2-0.4)	0.2	0.391
αβ CD8+DR+CD38+ T	13.5 (8.2-18.8)	19.2 (3.7-34.6)	12.1 (6.2-18.1)	7.0	0.130
αβ CD8+DR+CD38+ T (% of CD8 αβ T cells)	2.7 (1.9-3.4)	3.2 (0.8-5.5)	2.5 (1.7-3.3)	0.6	0.478
αβ CD8+CD38+CD69+ T	3.8 (1.5-6.1)	9.0 (-3.6-21.5)	2.6 (1.3-3.9)	6.4	0.105
αβ CD8+CD38+CD69+ (% of CD8 αβ T cells)	0.7 (0.4-0.9)	0.9 (0.4-1.4)	0.6 (0.3-0.9)	0.3	0.095
**NK IMMUNOPHENOTYPE (% OF TOTAL NK CELLS)**					
NK CD56^{dim}	157.3 (108.4-206.2)	117 (28-206)	167 (108-226)	-50	0.715
NK CD56^{dim} %	90.6 (86.4-94.8)	83.7 (57-111)	92.2 (91-94)	-8.5	0.751
NK CD56^{bright}	10.3 (7.8-12.8)	7.2 (4.2-10.2)	11.0 (8.0-14.1)	-3.8	0.391
NK CD56^{bright} %	7.31 (6.11-8.51)	5.6 (3.7-7.5)	7.7 (6.3-9.1)	-2.2	0.208
NK DR+	16.7 (11.8-21.5)	10.4 (3.8-16.9)	18.2 (12.4-24.0)	-7.8	0.268
NK DR+ %	13.2 (8.8-17.6)	8.0 (3.6-12.4)	14.4 (9.1-19.8)	-6.4	0.247
NK CD69+	9.5 (4.8-14.1)	5.4 (0.8-10.0)	10.4 (4.7-16.2)	-5.0	0.608
NK CD69+ %	6.7 (3.4-10.0)	3.5 (1.0-6.0)	7.5 (3.5-11.5)	-4.0	0.751
NK DR+CD69+	2.7 (-0.2-5.5)	1.0 (0.1-1.8)	3.1 (-0.5-6.6)	-2.1	0.679
NK DR+CD69+ %	2.8 (-0.3-5.8)	0.7 (0.01-1.4)	3.3 (-0.5-7.1)	-2.6	0.478
**PUTATIVE LYMPHOID PRECURSORS**					
PUTATIVE CD56-CD16+ PRECURSORS	11.3 (7.5-15.1)	5.7 (-1.6-13.0)	12.7 (8.2-17.1)	-6.9	0.060
PUTATIVE CD56-CD16+ PRECURSORS%	3.45 (2.23-4.67)	2.9 (-2.2-8.1)	3.6 (2.4-4.8)	-0.7	0.227
LIN-DNAM^{bright}	9.99 (6.56-13.41)	4.4 (-0.13-9.0)	11.3 (7.3-15.4)	-6.9	0.075
LIN-DNAM^{bright} %	3.06 (1.85-4.27)	1.6 (0.2-3.0)	3.3 (1.9b -4.8)	-1.79	0.291

Quantitative variables were expressed as means with a 95% confidence interval, while qualitative variables were represented as absolute frequencies with percentages relative to the total of the respective column. OR: odds ratio; MD: mean difference; **P** values were calculated by either unpaired two-tailed Student's t-test or Mann-Whitney u test according to variable distribution and sample size. DN: double negative; Univar.: univariate analysis.

Table S4. Comparison between CVID patients with active CMV/EBV replication vs non-replicating

	Total (31)	CMV/EBV (8)	No-CMV/EBV (23)	OR/ MD	P value (univ.)
**SAMPLE DEMOGRAPHICS**					
Male	14(45%)	3(38%)	11(48%)	0.6545	0.6980
Female	17(55%)	5(63%)	12(52%)	1.528	0.6980
Age	50.4 (44.1-56.7)	52.4 (38.4-66.3)	49.7 (842.1-57.3)	2.64	0.7142
**CLINICAL VARIABLES**					
Age at diagnosis	39.0 (32.3-45.8)	40.0 (25.1-54.9)	38.7 (30.5-46.9)	1.304	0.7820
Disease duration	13.7 (8.8-18.5)	13.6 (6.1-21.2)	13.7 (7.4-19.9)	-0.03	0.5265
Ig replacement therapy (IGRT)	26(84%)	7(88%)	19(83%)	1.474	>0.9999
IGRT duration	8.1 (4.8-11.4)	9.6 (2.2-17.0)	7.6 (3.6-11.5)	2.02	0.5205
Infections	29(94%)	8(100%)	21(91%)	Infinity	>0.9999
Autoimmunity	19(61%)	5(63%)	14(61%)	1.071	>0.9999
Splenomegaly	12(39%)	6(75%)	6(26%)	8.500	**0.0316**
Granulomatosis	6(19%)	2(25%)	4(17%)	1.583	0.6344
Malignancies	6(19%)	2(25%)	4(17%)	1.583	0.6344
Lymphadenomegaly	12(39%)	5(63%)	7(30%)	3.810	0.2055
Lung involvement	13(42%)	5(63%)	8(35%)	3.125	0.2285
Atopy	10(32%)	0(0%)	10(43%)	0	**0.0317**
**IMMUNOGLOBULINS**					
IgG (onset) (g/L)	3.1 (2.3-4.0)	3.1 (1.1-5.0)	3.2 (2.2-4.2)	-0.10	0.9205
IgA (g/L)	0.48 (-0.02-0.18)	0.26 (-0.01-0.53)	0.56 (-0.12-1.24)	-0.30	>0.9999
IgM (g/L)	0.38 (0.18-0.59)	0.28 (-0.05-0.60)	0.42 (0.15-0.69)	-0.14	0.5420
IgE (kU/L)	10.1 (2.7-17.5)	6.5 (-5.57-18.62)	11.6 (1.8-21.5)	-5.1	0.3650
**GENERAL IMMUNOPHENOTYPE (ABSOLUTE COUNT: CELLS/ µL)**					
Lymphocytes	2118 (1698-2538)	2075 (963-3187)	2133 (1654-2612)	-59	0.520
Lymphocytes (% of WBC)	35.3 (31.2-39.5)	36.5 (26.3-48.8)	35.0 (30.5-39.5)	1.5	0.642
Monocytes	369 (306-432)	368 (194-542)	370 (300-440)	-2	0.981
Monocytes (% of WBC)	6.3 (5.3-7.2)	6.1 (5.2-7.1)	6.3 (5.0-7.7)	-0.3	0.674
T cells	1446 (1177-1716)	1533 (727-2338)	1417 (1130-1703)	116	1.000
T Lymphocytes (% of Lymphocytes)	69.3 (65.4-73.2)	74.2 (68.3-80.0)	67.6 (62.7-72.5)	6.6	0.132
B cells	266 (155-378)	209 (-17-435)	277 (143-410)	-68	0.464
B Lymphocytes %	10.9 (7.9-13.8)	8.3 (4.2-12.5)	11.7 (8.0-15.6)	-3.4	0.464
NK cells	172 (121-222)	124 (54-194)	188 (123-253)	-64	0.520
NK cells (% of Lymphocytes)	8.6 (6.5-10.7)	7.0 (3.1-10.9)	9.2 (6.6-11.7)	-2.1	0.411
NKT-LIKE	58 (31-85)	72 (-13-156)	53 (26-80)	19	0.808
NKT-LIKE cells (% of Lymphocytes)	2.7 (1.8-3.6)	2.7 (1.0-4.4)	2.7 (1.5-3.9)	0.02	0.740
Lineage negative (Lin-)	173 (134-211)	169 (83-256)	174 (128-220)	-4	0.842
Lin- cells (% of lymphocytes)	9.4 (7.3-11.5)	9.6 (5.5.13.8)	9.3 (6.8-11.9)	0.3	0.707
**B CELLS IMMUNOPHENOTYPE (% OF TOTAL B CELLS)**					

Naïve	54.4	60.7	52.5	8.2	0.5234
$IgM^{+}IgD^{++}CD38^{+}CD27^{-}CD21^{+}$	(46.3-62.6)	(48.3-73.0)	(42.1-62.8)		
CD21low	9.4	11.1	8.8	2.3	0.5569
$IgM^{+}IgD^{+}CD38^{low}CD27^{-}CD21^{low}$	(5.3-13.5)	(-0.6-22.9)	(4.3-13.3)		
Class-switched memory	4.1	2.0	4.9	-2.9	0.9735
$IgM^{-}IgD^{-}CD38^{+}CD27^{+}CD21^{+}$	(1.6-6.6)	(0.6-3.3)	(1.6-8.2)		
Non-switched memory	8.3	3.2	10.0	-6.8	0.4433
$IgM^{++}IgD^{+}CD38^{+}CD27^{+}CD21^{+}$	(4.1-12.5)	(1.6-4.8)	(4.5-15.6)		
Transitional	7.6	7.4	7.7	-0.3	0.0845
$IgM^{++}IgD^{++}CD38^{++}CD27^{-}CD21^{+}$	(3.2-12.0)	(3.1-11.7)	(1.6-13.7)		
Plasmablasts	0.37	0.66	0.26	0.40	0.1648
$IgM^{-/+}IgD^{-}CD38^{+++}CD27^{++}CD21^{+}$	(0.05-0.69)	(-0.38-1.71)	(-0.04-0.57)		
**T CELLS IMMUNOPHENOTYPE**					
αβ T cells	1409	1503	1376	128	1.000
	(1145-1673)	(701-2307)	(1100-1653)		
αβ T cells	97.4	98.0	97.3	0.7	0.172
(% of T cells)	(96.8-98.1)	(96.6-99.3)	(96.5-98.0)		
γδ T cells	37.1	28.7	40.1	-11	0.492
	(24.8-49.4)	(7.9-49.5)	(24.4-55.7)		
γδ T cells	2.6	2.0	2.7	-0.7	0.172
(% of T cells)	(1.9-3.2)	(0.7-3.4)	(2.0-3.5)		
αβ CD4+ T	805	738	828	-90	0.566
	(667-942)	(448-1027)	(659-997)		
αβ CD4+ T	58.7	53.0	60.8	-7.8	0.219
(% of αβ T cells)	(54.4-63.2)	(40.5-65.4)	(56.2-65.4)		
αβ CD8+ T	528	679	476	203	0.437
	(384-672)	(172-1186)	(352-600)		
αβ CD8+ T	35.7	41.3	33.7	7.7	0.121
(% of αβ T cells)	(31.6-39.7)	(30.8-51.9)	(29.4-38.0)		
αβ DN T cells	68.0	73.3	66.1	7.2	0.947
	(48.5-87.4)	(28.7-117.9)	(42.8-89.4)		
αβ DN T cells	5.0	5.1	5.0	0.1	0.877
(% of αβ T cells)	(3.9-6.1)	(2.2-8.0)	(3.8-6.2)		
αβ CD4+CD8+ T	8.5	13.9	6.6	7.3	1.000
	(2.6-14.3)	(-9.8-37.5)	(2.8-10.5)		
αβ CD4+CD8+ T	0.52	0.56	0.51	0.05	0.611
(% of αβ T cells)	(0.24-0.80)	(-0.02-1.15)	(0.16-0.86)		
αβ CD4+DR+ T	36.1	33.8	36.9	-3.1	0.912
	(27.6-44.5)	(17.0-50.6)	(26.3-47.4)		
αβ CD4+DR+ T	5.1	5.4	5.0	0.5	0.740
(% of CD4 αβ T cells)	(3.9-6.2)	(2.6-8.2)	(3.6-6.3)		
αβ CD4+CD69+ T	11.6	9.6	12.3	-2.7	0.707
	(8.2-15.1)	(3.9-15.3)	(7.9-16.8)		
αβ CD4+CD69+ T	1.6	1.5	1.6	-0.1	0.707
(% of CD4 αβ T cells)	(1.0-2.1)	(0.7-2.3)	(0.9-2.3)		
αβ CD4+DR+CD69+ T	1.1	1.3	1.0	0.3	0.912
	(0.6-1.6)	(-0.3-2.8)	(0.5-1.5)		
αβ CD4+DR+CD69+ T	0.2	0.20	0.14	0.06	0.437
% of CD4 αβ T cells	(0.1-0.2	(-0.01-0.41)	(0.06-0.21)		
αβ CD4+DR+CD38+ T	13.8	19.1	12.0	7.2	0.121
	(8.9-18.8)	(6.6-31.7)	(6.4-17.5)		
αβ CD4+DR+CD38+ T	2.1	3.09	1.69	1.40	0.187
(% of CD4 αβ T cells)	(1.4-2.8)	(1.13-5.05)	(0.98-2.39)		
αβ CD4+CD38+CD69+ T	4.6	5.3	4.4	0.9	0.386
	(3.0-6.3)	(1.4-9.2)	(2.4-6.3)		
αβ CD4+CD38+CD69+ T	0.6	0.73	0.57	0.16	0.339
(% of CD4 αβ T cells)	(0.4-0.8)	(0.35-1.10)	(0.37-0.76)		
αβ CD8+DR+ T	32.2	37.9	30.2	7.7	0.145
	(22.1-42.3)	(22.1-53.7)	(17.2-43.2)		
αβ CD8+DR+ T	6.9	7.94	6.50	1.44	0.386
(% of CD8 αβ T cells)	(5.3-8.4)	(4.01-1.87)	(4.72-8.29)		
αβ CD8+CD69+ T	22.1	22.7	21.9	0.8	0.295
	(6.7-37.6)	(2.3-43.2)	(1.5-42.3)		
αβ CD8+CD69+ T	4.3	3.28	4.63	-1.35	0.091
(% of CD8 αβ T cells)	(1.3-7.3)	(2.65-3.90)	(0.52-8.74)		
αβ CD8+DR+CD69+ T	1.6	2.2	1.4	0.8	0.187
	(1.1-2.1)	(0.6-3.7)	(0.9-2.0)		

αβ CD8+DR+CD69+ T (% of CD8 αβ T cells)	0.4 (0.3-0.5)	0.49 (0.17-0.80)	0.32 (0.21-0.43)	0.16	0.275
αβ CD8+DR+CD38+ T	13.5 (8.2-18.8)	18.3 (6.7-29.9)	11.9 (5.5-18.2)	6.4	0.082
αβ CD8+DR+CD38+ T (% of CD8 αβ T cells)	2.7 (1.9-3.4)	3.46 (1.28-5.63)	2.38 (1.64-3.11)	1.08	0.295
αβ CD8+CD38+CD69+ T	3.8 (1.5-6.1)	7.1 (-1.8-16.1)	2.6 (1.2-4.1)	4.5	0.158
αβ CD8+CD38+CD69+ (% of CD8 αβ T cells)	0.7 (0.4-0.9)	0.77 (0.38-1.16)	0.62 (0.31-0.93)	0.15	0.121
**NK IMMUNOPHENOTYPE (% OF TOTAL NK CELLS)**					
NK CD56^{dim}	157.3 (108.4-206.2)	102.0 (36.4-167.6)	176.6 (114.3-238.9)	-74.6	0.317
NK CD56^{dim} %	90.6 (86.4-94.8)	85.8 (67-4-104.2)	92.2 (90.7-93.7)	-6.4	0.877
NK CD56^{bright}	10.3 (7.8-12.8)	6.5 (4.1-8.8)	11.6 (8.4-14.8)	-5.2	0.082
NK CD56^{bright} %	7.31 (6.11-8.51)	6.2 (4.4-7.9)	7.7 (6.2-9.2)	-1.6	0.362
NK DR+	16.7 (11.8-21.5)	9.4 (4.7-14.2)	19.2 (13.0-25.4)	-9.8	0.074
NK DR+ %	13.2 (8.8-17.6)	9.0 (5.5-12.4)	14.7 (8.8-20.5)	-5.7	0.492
NK CD69+	9.5 (4.8-14.1)	4.8 (1.5-8.0)	11.1 (4.9-17.3)	-6.4	0.464
NK CD69+ %	6.7 (3.4-10.0)	4.2 (1.7-6.7)	7.6 (3.2-12.0)	-3.4	0.947
NK DR+CD69+	2.7 (-0.2-5.5)	0.8 (0.1-1.4)	3.3 (-0.6-7.2)	-2.5	0.674
NK DR+CD69+ %	2.8 (-0.3-5.8)	0.6 (0.1-1.2)	3.5 (-0.6-7.7)	-2.9	0.642
**PUTATIVE LYMPHOID PRECURSORS**					
PUTATIVE CD56-CD16+ PRECURSORS	11.3 (7.5-15.1)	5.10 (0.07-10.12)	13.47 (8.81-18.13)	-8.4	**0.012**
PUTATIVE CD56-CD16+ PRECURSORS%	3.45 (2.23-4.67)	2.65 (-0.89-6.19)	3.73 (2.43-5.02)	-1.07	0.145
LIN-DNAM^{bright}	9.99 (6.56-13.41)	3.90 (0.72-7.07)	12.10 (7.86-16.34)	-8.2	**0.034**
LIN-DNAM^{bright} %	3.06 (1.85-4.27)	1.51 (0.53-2.50)	3.60 (2.02-5.17)	-2.09	0.172

Quantitative variables were expressed as means with a 95% confidence interval, while qualitative variables were represented as absolute frequencies with percentages relative to the total of the respective column. P values were calculated by either unpaired two-tailed Student's t-test or Mann-Whitney u test according to variable distribution and sample size. OR: odds ratio; MD: mean difference; DN: double negative; Univ.: univariate analysis. MAPO

Supplementary figure

Fig. S1

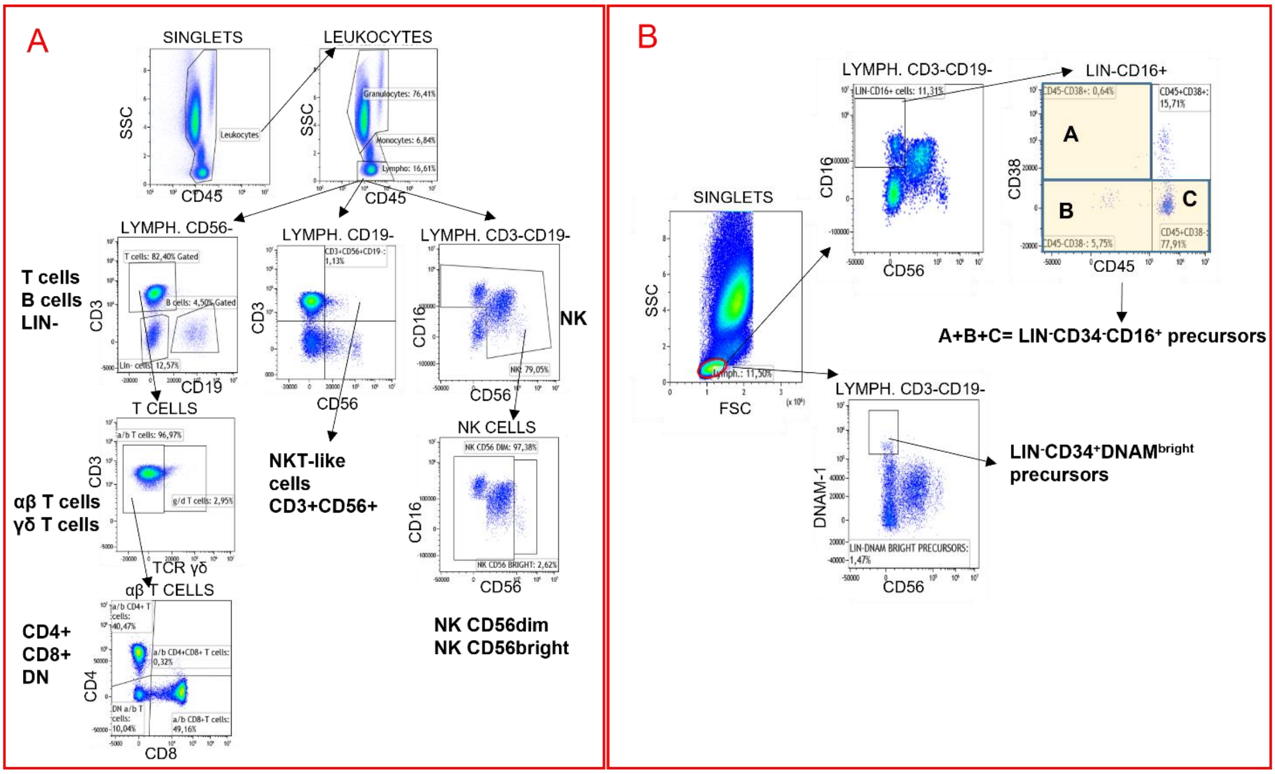

Legend to supplementary figure

Fig. S1: Gating strategy. A. Leukocyte population gating strategy (CD45/SSc). **B**. Side Scatter/Forward Scatter gating strategy to identify inflammatory lymphoid precursors (LIN-CD34-CD16+, LIN-CD34+DNAMbright). Abbreviations: DN: double negative; FSC: forward scatter; LIN: lineage; Lymph.: lymphocytes; SSC: side scatter.
